# Nutraceuticals: A Promising Therapeutic Approach in Ophthalmology

**DOI:** 10.3390/nu14235014

**Published:** 2022-11-25

**Authors:** Carlos Rodrigo Castro-Castaneda, Francisco Altamirano-Lamarque, Alan Gabriel Ortega-Macías, Francisco J. Santa Cruz-Pavlovich, Alejandro Gonzalez-De la Rosa, Juan Armendariz-Borunda, Arturo Santos, Jose Navarro-Partida

**Affiliations:** 1 Tecnologico de Monterrey, Escuela de Medicina y Ciencias de la Salud, Campus Guadalajara, Zapopan 45138, Mexico; 2 Centro de Retina Medica y Quirurgica, S.C., Hospital Puerta de Hierro, Zapopan 45116, Mexico; 3 Department of Molecular Biology and Genomics, Institute for Molecular Biology and Gene Therapy, University of Guadalajara, Guadalajara 44340, Mexico

**Keywords:** nutraceutical, oxidative stress, antioxidant, ophthalmic diseases

## Abstract

Oxidative stress represents one of the main factors driving the pathophysiology of multiple ophthalmic conditions including presbyopia, cataracts, dry eye disease (DED), glaucoma, age-related macular degeneration (AMD) and diabetic retinopathy (DR). Currently, different studies have demonstrated the role of orally administered nutraceuticals in these diseases. For instance, they have demonstrated to improve lens accommodation in presbyopia, reduce protein aggregation in cataracts, ameliorate tear film stability, break up time, and tear production in dry eye, and participate in the avoidance of retinal neuronal damage and a decrease in intraocular pressure in glaucoma, contribute to the delayed progression of AMD, or in the prevention or treatment of neuronal death in diabetic retinopathy. In this review, we summarized the nutraceuticals which have presented a positive impact in ocular disorders, emphasizing the clinical assays. The characteristics of the different types of nutraceuticals are specified along with the nutraceutical concentration used to achieve a therapeutic outcome in ocular diseases.

## 1. Introduction

Oxidative stress has been demonstrated to take part in a wide variety of ocular diseases including dry eye disease (DED), glaucoma, age-related macular degeneration (AMD), diabetic retinopathy (DR), among others. Reactive oxygen species (ROS), either from endogenous or exogenous sources, have shown to be key players in cell survival of ocular tissues [1]. For that reason, the eye has become a specific target for new medical approaches based on different foods and specific nutrients with antioxidant capabilities [2,3,4]. Importantly, the implementation of these products has demonstrated to influence ocular health and has turned around the premise from offering drugs with increased adverse effects to the possibility of using more natural extracts to achieve better patient care.

Currently, the vast terminology used to describe the food and its components with therapeutic activities has presented certain difficulties in establishing specific differences between terms including functional foods, supplements, and nutraceuticals.

The term “functional food” comes from Japanese roots, where it was acknowledged as a food that could have an impact beyond gastronomic pleasure and energetic supply. Currently, this term is specified as a food capable of enhancing physiological functions and preventing or even curing diseases apart from its nutritional value but not being essential for life. In order to reach consensus, the Japanese criteria Foods for Specified Health Uses (FOSHU) for functional foods were developed and included three main characteristics: to not be presented as pills, capsules or powder but as natural food, to be included in everyday diet and, lastly, to have a functional impact in human physiology, including complaint control, immunological improvement, aging delay and prevention and recovery from specific pathologies [5,6,7].

Conversely, according to Johns Hopkins Bloomberg School of Public Health, supplements are a concentrate, metabolite, constituent, extract or a combination of vitamins, minerals, amino acids, herbs, botanicals, and nutraceuticals. These products, in contrast to functional foods, are intended to be ingested in their pharmacological configuration including capsules, tablets, pills, powder, softgel, or gelcap. Additionally, supplements have shown not to be represented as regular or as conventional food [7,8].

Lastly, nutraceuticals can be defined as supplements originating from food that contains a bioactive agent in a greater concentration than in a balanced diet or in a concentration equivalent to it, which functions as an enhancement for pharmacological treatments, or for the delay, improvement or even prevention of diseases [7].

Functional foods and supplements, especially nutraceuticals, have shown to possess therapeutic activity in preclinical studies regarding ophthalmic disorders. For instance, Nagashima et al. have determined in rat models the effect of *Enterococcus faecium WB2000, Lactobacillus pentosus TJ515* and resveratrol on lens elasticity after its oral administration. Briefly, an overall improvement in the lens elasticity was noted in both long-term effects (40 weeks) using 0.042, 0.007 and 0.088 mg/day of these supplements, respectively, and in the short term (4 weeks) administering 0.21, 0.007, 0.44 mg/day, respectively. These findings have established a potential approach for managing the progression of near vision impairment [9]. Moreover, topical pirenoxine has been demonstrated to significantly increase lens elasticity by suppressing the hardening of the lens in rat models with presbyopia induced by tobacco smoke [10].

Conversely, vitamin C has shown to play an important protective role against cataracts [10]. Authors have evidenced lesser corneal opacities after 1.5 g/L of vitamin C administered to SMP30/GNL knockout mice, which are unable to synthesize vitamin C [11]. Moreover, Blondin et al. have demonstrated less protein damage induced by UV light exposure after including vitamin C (50 mg/day) in the guinea pigs’ diet. These authors defined damage by measuring an increase in aggregates and attenuation of activity of the exopeptidase [12]. Lastly, vitamin C (50 mg), along with other vitamins, has proved to influence oxidative stress of the lens by increasing the activity of glutathione peroxidase and decreasing peroxidation in diabetic rats induced by streptozotocin [13].

On the other hand, *Curcuma longa*, also known as curcumin, has demonstrated in vitro an anti-inflammatory effect, which is beneficial to ocular surface pathologies such as dry eye. Corneal epithelial cells were cultured in a hyperosmotic medium to be exposed to curcumin at doses of 5µM. The results have shown that curcumin is capable of reducing the expression of proinflammatory cytokines, including interleukin-1β (IL-1β), interleukin-6 (IL-6) and tumor necrosis factor- alpha (TNF-a), involved in DED pathophysiology [14]. Similarly, in vitro induced cytotoxic human epithelial cells were used to demonstrate the effects of the organic compounds of the plant *Polygonum cuspidatum (PCE),* including anthraquinones, resveratrol, flavonoids and polydatin. Their anti-inflammatory effects were demonstrated after exposure to different PCE doses (10, 100 or 250 mg/kg), mainly by an inhibition of the expression of IL-6, TNF-a, and cyclooxygenase-2 (COX-2) and activation of nuclear factor kappa B (NF-kB) [15]. Additionally, it was evidenced an amelioration of symptoms and tear film parameters such as Schirmer’s test score, tear break-up time (TBUT) and keratoconjunctival fluorescein staining in rat models of DED after the oral administration of goji berry at either low (250 mg/kg/bw), medium (350 mg/kg/bw) or high (500 mg/kg/bw) dose in their diet [6,16]. Moreover, different studies have demonstrated an improvement in tear volume and mucin 4 (MUC4) expression after orally administering PCE in rat models [15]. Lastly, the use of a topical combination of 3% diquafosol sodium and vitamin E at different doses (0.005% or 0.01%) has demonstrated an amelioration of the ocular surface inflammation, the density of goblet cells in the conjunctiva, the lipid layer of the tear film and, consequently, an improvement in TBUT and fluorescein stain scores in induced DED mouse models. This suggests the potential benefit of vitamin E in the treatment of DED [17].

Conversely, Kamalden et al. have shown a counteraction of ischemic consequences to the retina after an increase in the intraocular pressure (IOP) using intraperitoneal genistein (10 mg/kg) in rats. In this study, genistein has demonstrated to limit neuronal damage and oxidative response in the eye induced by the rising of the IOP [18]. Additionally, Davis et al. have reported the influence and neuroprotective function of curcumin against the toxicity induced by glutamate and hypoxia, induced by cobalt chloride in vitro R28 retinal precursor cells. These authors implemented a topical nanocarrier for the transport of curcumin (4.3 mg/mL) to demonstrate its effect in rat models with ocular hypertension. This research study resulted in a significant reduction in the loss of retinal ganglion cells [19].

In an in vitro study, Chichili et al. evidenced the plausible antioxidant effects of beta-carotene in AMD. Incubating retinal pigment epithelium cells (ARPE-19 cells) in a beta-carotene rich concentration (1 µM) obtained from tomatoes combined with lutein and lycopene resulted in a reduction in and protection against oxidative stress [20]. Moreover, Bhatt et al. have determined the anti-inflammatory and antioxidant properties of resveratrol PLGA nanoparticles (10 μm) in ARPE-19 cell culture, especially by displaying an anti-angiogenic property through the inhibition of VEGF expression [21].

Lastly, diabetic retinopathy (DR) has also been benefited using nutraceuticals. Preclinical studies in diabetic-induced mice have shown to prevent the generation of ROS and to improve visual function by suppressing the visual impairment induced by diabetes and determined by electroretinograms after adding lutein (0.1%) to their diet [22]. Furthermore, Kowluru et al. have evidenced in rat models an improvement in the increment of VEGF induced by diabetes, and a reduction in proinflammatory cytokines in the retina after giving them a powder diet, which included eicosapentaenoic acid (EPA; 650 mg), α lipoic acid (750 mg), vitamin C (300 mg), benfotiamine (1 g), vitamin D3 (10,000 IU), fish oil EE70% (1.6 g), vitamin E (300 IU), docosahexaenoic acid (DHA; 500 mg), lutein (20 mg), tocomin (200 mg), zeaxanthin (40 mg) and a registered blend with resveratrol and curcumoids [23].

Due to the increasing preclinical investigation on food and its components in ophthalmology and the lack of compiled clinical information, we describe the applications of functional foods and supplements, particularly nutraceuticals, in ocular health research, emphasizing the clinical assays. We specify the characteristics of the different types of nutraceuticals along with the nutraceutical concentration used to achieve a therapeutic outcome in the main ocular diseases.

## 2. Functional Foods

As previously explained, functional foods are defined as food capable of enhancing physiological functions and preventing or even curing diseases apart from its nutritional value. The role of functional food in human physiology and the immune system has shown to be secondary to its specific components including probiotics, prebiotics, micronutrients, or even a combination of the first two, known as synbiotics. Probiotics such as *Lactobacilli* and *Bifidobacteria* are defined as living microorganisms that offer beneficial effects to the host, whereas prebiotics, including inulin and lactulose, present a positive influence on the host’s microbiota. Finally, micronutrients are elements with specific biologic actions essential to cellular function and are composed of minerals, vitamins, and fatty acids [5,24]. All these components have been demonstrated to participate in the preservation of health and in the therapeutics or prevention of different pathologies, including ocular diseases [6,24,25,26,27,28,29].

Conventional fruits and vegetables can be used as functional foods. It has been described that the regular consumption of fruits and vegetables is able to decrease the risk of developing glaucoma, particularly the consumption of green collard and kale, carrots and peaches which are associated with decreased odds of glaucoma risk of 69%, 64% and 47%, respectively [30,31].

Despite the positive effects of functional foods on overall health and ocular health, the main problem with these products has been proved to be their lack of regulation, lessening the information regarding their quality process, the type, and the exact amount of these foods. Hence, a significant number of adverse effects have been related to functional foods in the peer-reviewed literature such as the risk of allergic reactions or anaphylaxis [32]. For example, functional foods with high levels of honey and salicylates have provoked gastrointestinal and respiratory symptoms including nausea, vomiting or hyperventilation in susceptible people [33,34]. Lastly, plant-derived functional foods, such as *St. John’s wort*, have been demonstrated to modify drug pharmacodynamics and decrease drug bioavailability [33,35]. By contrast, nutraceuticals have demonstrated the advantage of being products that undergo a quality surveillance and, consequently, acquire an exact name and amount of their ingredients, leading to a significant decrease in the incidence of adverse effects.

## 3. Supplements and Nutraceuticals

Compared to functional foods, supplements are described as complements of the diet ingested in their pharmacological configuration including capsules, tablets, pills, powder, softgel, or gelcap, which are not represented as regular or as conventional food. Supplements can be divided into many subgroups, such as concentrates, metabolites, constituents, extracts, nutraceuticals or a combination of vitamins, minerals, amino acids, herbs, and botanicals. The most popular reason for including supplements in the American diet is for overall health, presenting an estimated consumption percentage of 70% of these products. The most recognized and studied supplements include omega 3/fish oil, multivitamin/mineral supplements, calcium supplements and nutraceuticals [7,8,36]. Nutraceuticals could be the most important of all supplements in ocular health, due to their proven therapeutic capability in animal models and clinical trials. These products have proved an easy extraction and application, as well as a vast functionality in the human body [7,37,38]. In the following paragraphs, the classification of nutraceuticals will be described and their applications in ophthalmology posteriorly exemplified.

## 4. Classification of Nutraceuticals

Along with the established differences between functional foods and supplements, it is relevant to highlight the classification of nutraceuticals for a better understanding. Nutraceuticals can be classified depending on their chemical nature (lipidic, proteic, as a carbohydrate, micronutrient or microbial), mechanism of action (based on physiologic or metabolic impact) or even by their origin (plant or animal-derived and microbial origin) [39]. Furthermore, other authors have classified nutraceuticals into two main categories: traditional or non-traditional.

### 4.1. Traditional and Non-Traditional Nutraceuticals

Traditional nutraceuticals have shown the characteristic of being obtained from microbial, animal, plant, or mineral sources, granting their name of natural nutraceuticals. These nutraceuticals are composed of three primary groups, including chemical components, enzymes, or probiotics from which different subgroups arise. Phytochemicals, nutrients, and herbals represent the subcategories belonging to the chemical components [40,41].

On the other hand, non-traditional nutraceuticals, which are the other fundamental group, present a different preparation using biotechnology and are well-known as artificial or unnatural nutraceuticals. Two main groups have been identified in their classification: the recombinant and the food fortified with nutraceuticals [40,41].

#### 4.1.1. Traditional Nutraceuticals: Chemical Components

##### Phytochemical Nutraceuticals

Phytochemical nutraceuticals are characterized by the presence of plant chemicals, which have demonstrated a specific action in human metabolic, physiologic, or immunologic processes. Although there is a wide variety of phytochemicals, some of them have demonstrated an impact in ocular, neurodegenerative and even psychiatric diseases, including polyphenols (flavonoids and non-flavonoids), carotenoids, quinones, organosulfur compounds, saponins and alkaloids [40,41,42,43].

1. Phytochemical nutraceuticals: Polyphenols

Flavonoids polyphenols are compounds which can be obtained from a wide variety of sources including vegetables, berries, legumes or fruits [41]. Chemically, flavonoids are formed mainly through oxidation and hydroxylation process variations in the central pyran ring (ring C), from which the phenolic rings (ring A and B) are attached and give rise to an incredible diversification of flavonoids, including isoflavones, flavanols, anthocyanins, flavanones, anthocyanidines, among others [44,45]. Their main effects have been demonstrated mostly as antioxidants, but also in the prevention of different malignancies, such as prostate or breast cancer, and diabetes mellitus regulation [41,43,46,47]. Additionally, flavonoids have proved to exert antidepressant effects by regulating neurotransmitter receptors and attenuating serotonin, norepinephrine, 5-hydroxyindoleacetic acid (5-HIAA), and dopamine [48,49]. Similarly, these compounds have benefited neuroinflammation secondary to ischemia-reperfusion injuries or neurodegenerative diseases through the regulation of transcription factors, signaling pathways, gene expression or avoidance of an increase in neurotoxic mediators such as prostaglandin E2 (PGE2) [42,45].

Furthermore, polyphenols also include the non-flavonoids compounds. Similarly to flavonoids, these compounds can be obtained from berries, but raisins, peanuts, dark grapes, or even turmeric roots represent additional sources [41]. Moreover, they have presented an aromatic ring as their basic structure along with hydroxyl groups, which change in every subgroup. One of the main subgroups of non-flavonoids are the phenolic acids, which have shown two skeletons: the hydroxybenzoic and the hydroxycinnamic acid. The former one includes the p-hydroxybenzoic, vanillic, gallic, syringic and protocatechuic acid, whereas the latter includes sinapic, p-coumaric, ferulic and caffeic acids [44]. Both subgroups have demonstrated a positive effect in several diseases. For instance, Verma et al. have evidenced that gallic acid presents an inhibitory action against carcinogenesis. The pathways involved in the process are the ATM kinase activation, induction of intrinsic (Cytochrome c) or extrinsic (Fas/FasL) apoptosis pathway and mitochondrial dysfunction, among others, all of which lead to cell death [50]. Moreover, syringic acid have demonstrated cardiovascular benefits after myocardial ischemia, mainly by reducing the injury induced by reperfusion. This was achieved through the activation of the PI3K/Akt/GSK-3β pathway, resulting in a decrease in the size of the infarct, the apoptosis induced by the mitochondria and, consequently, the levels of CK-MB and LDH [51]. On the other side, other non-flavonoid polyphenols such as ferulic and caffeic acids have been demonstrated to avoid cardiotoxicity or have a direct cardioprotection, antioxidant or antithrombotic effect, and reduction in risk factors such as hyperlipidemia or diabetes [52,53,54,55]. Other studies have shown their possible applications in lung injury, diabetes, liver fibrosis or even in dermatologic diseases [56,57,58,59].

2. Phytochemical nutraceuticals: Carotenoids

Additionally, carotenoids are traditional nutraceuticals also classified inside the group of phytochemicals. These compounds have been presented to be available in the diet, especially from vegetables, fruits, and egg yolk [41]. Characteristically, carotenoids have shown a carbon backbone made up of 40 carbons and 8 isoprene molecules. Two groups are further distinguished regarding the presence of hydrogen and carbon molecules referred as carotenes, which include α-carotene, β-carotene, and lycopene, or by the presence of oxygen in the terminal ring referred to as xanthophylls such as lutein, cryptoxanthin, and zeaxanthin. Many authors have proved their effects in human physiology, mainly as antioxidants [60,61,62]. Due to their capacity to produce vitamin A after several enzymatic cleavages, they can also present provitamin A activity, including α-carotene, β-carotene and cryptoxanthin, whereas zeaxanthin, lycopene and lutein do not [62]. Several authors have demonstrated the antioxidant ability of β-carotene not only in the reduction in bladder, esophageal or breast cancer risk, but also against kidney injury induced by thioacetamide in rats, mainly through a decreased production of angiotensin-converting enzyme (ACE) and an increase in superoxide dismutase (SOD), catalase (CAT) and glutathione peroxidase (GSH) [63,64]. Compared to β-carotene, lycopene has shown greater benefits in the prevention of the adverse effects after chemotherapy as well as in the lowering of blood pressure and LDL cholesterol [63]. On the other side, a positive influence regarding the use of lutein has been demonstrated in the evolution of breast cancer via inhibitory growth mechanisms, where the expression of heme oxygenase-1 and SOD-2 along with survival proteins and nuclear factor-kB have shown to be restrained, resulting in apoptosis of malignant cells [65]. Interestingly, both lycopene and lutein have also shown to reduce the risk of gastrointestinal, prostate or even ovarian cancer in a significant manner [60,63].

3. Phytochemical nutraceuticals: Quinones

Furthermore, quinones are phytochemical nutraceuticals obtained from plants including *Rubiaceae, Rhamnaceae*, and *Fabaceae*. Quinones are chemically characterized by a cyclic diketone structure. One of the most described quinone subgroups are the anthraquinones, which include emodin, cascarin, chrysophanol, catenarin and rhein. Moreover, anthraquinones are divided into monomers or bianthraquinones depending on their nucleus structure [66,67,68]. Several authors have described the biological activities of anthraquinones including anti-inflammatory, antimicrobial, anticancer and laxative properties [66,67]. The inhibition of proinflammatory mediators, such as TNF-a and IL-6, and the downregulation of kinases including PTK, PKC, and CaMPKS, has been demonstrated by emodin, chrysophanol, and rhein anthraquinones. Additionally, emodin has proved to have a direct cytotoxic effect and to influence cell cycle, cell apoptosis and resistance of drugs of cancer cells, resulting in an antitumorigenic effect [66,68]. Antimicrobial properties have also been evidenced after the use of anthraquinones, mainly by protein synthesis and biofilm genes inhibition, as well as the destruction of cell membranes. Finally, antioxidant effects have been demonstrated by the inhibition of lipid peroxidation and by the scavenging of free radicals [68,69].

4. Phytochemical nutraceuticals: Organosulfur compounds

Other types of phytochemical nutraceuticals are the organosulfur compounds (OSCs) that are present in animals and plants, including vegetables of *Allium* group such as garlic, shallots, onions, or leeks and *Brassica* genus such as broccoli, cabbage, or cauliflower. OSCs can be divided structurally depending on the functional groups holding a sulfur, after which different compounds can be described including allicin, cysteine, dibenzothiophene, sulfanilamide, penicillin and lipoic acid, among others [70,71,72,73]. Generally, OSCs have demonstrated anti-inflammatory, antioxidant and antimicrobial abilities [71,74,75]. Those specific properties have been tested in different pathologies, resulting in positive results as a cardioprotective, antiviral or antitumor agent [70,74,76]. For instance, one of the most described sources of OSCs is garlic, also known as *Allium sativum.* Garlic has shown to be effective against herpes, influenza, coxsackie, and HIV virus [72,74]. Additionally, several randomized clinical trials have proved to decrease the severity and occurrence of flu and colds by improving immune T and NK cells after orally taking garlic extract (2.5 g/day) [77,78]. Interestingly, after implementing the use of garlic organosulfur compounds in an in silico trial, different researchers have demonstrated the potential benefits, especially of alliin, against COVID-19 after inhibiting the main protease of the virus and increasing the evidence of the use of natural compounds for antiviral properties [79,80].

5. Phytochemical nutraceuticals: Saponins

As well as the previous phytochemical nutraceuticals, saponins have been shown to produce different biological effects, including hypolipidemic, hypoglycemic, antiproliferative, and immunostimulant properties, inhibition of platelet aggregation, or hypercalciuria and acute lead poisoning treatment [81]. These nutraceuticals are glycosides with heat-stable and amphipathic properties, which are present in plants, especially legumes. Ultrastructurally, saponins consist of oligosaccharides moieties with a non-polar (sapogenin) and a polar (sugar) group bound to it, which confer these nutraceuticals an active surface responsible for their biological effects [82]. Two main types have been described: the protopanaxatriol and protopanaxadiol, the latter being the most effective against obesity. For instance, ginseng and soy saponins have demonstrated antitumor effects against several types of cancer, including the arrest of cell cycle in breast cancer cells and cytotoxic activity against cervical, fibrosarcoma and hepatocellular cancer cells. Furthermore, saponins have demonstrated an important influence in obesity and lipid metabolism, mainly by inhibiting adipogenesis through the activation of AMPK and the pancreatic lipase, preventing fat absorption [81]. Moreover, these nutraceuticals have shown to effectively reduce blood glucose levels through the improvement in insulin response mediated by PPAR-y, resulting in better glucose metabolism and an increase in plasma insulin levels and insulin release from the pancreas [81,83].

6. Phytochemical nutraceuticals: Alkaloids

Alkaloids represent the last group of phytochemical nutraceuticals which can be found in plants and bacteria. Characteristically, alkaloids present an alkali-like structure, and are found either as free bases or salts of organic acids. These nutraceuticals can be subclassified into three main categories based on their structure. First, true alkaloids present a precursor amino acid and possess a nitrogen atom in their heterocycle. Differing from true alkaloids, the proto- alkaloids lack the nitrogen atom. Finally, the pseudo alkaloids are non-heterocyclic with no amino acid precursor and present the nitrogen atom in the side chain [84]. These compounds have demonstrated to be very useful for cancer drugs such as vincristine, vinblastine and taxol, blood drugs such as vincamine, and antimalarial drugs including chloroquine and quinine [85]. Interestingly, different authors have reported the use of mushrooms containing alkaloids with cytotoxic and anti-tuberculosis activity [86,87].

##### Nutrients

Nutrients are included in the three main groups of the chemical compounds of the traditional nutraceuticals. These substances are obtained from animal products, vegetables, fruits and whole grain-cereals, among others. As previously well explained, fatty acids, vitamins, and minerals have shown a great impact on a wide variety of diseases, including cancers, cerebrovascular, ocular, or even neurodegenerative diseases, as well as in immunomodulation and inflammatory pathways [41,85].

1. Nutrients: Peptides and Bioactive Peptides

Chemically, the bioactive peptides are composed of amino acids joined by covalent bonds, either amide or peptide bonds. Bioactive peptides are defined as protein fragments with a positive impact on body functions which influence health. Their primary sources have been described to be from animal products such as eggs, milk (casein and whey) and meat. Nevertheless, plant sources including soy, oat and wheat have become great sources for obtaining these nutrients [88]. Bioactive peptides are classified based on their mechanism of action including immunomodulatory, antihypertensive, antimicrobial, opioid, antioxidant, or antithrombotic activity [89].

Importantly, their biologic antimicrobial and anti-inflammatory effects are presented after a proteolytic effect of intestinal bacteria (*Lactobacillus*) on the milk which results in the formation of bioactive peptides from lactoferrin. Furthermore, the bioactive peptides can inhibit angiotensin I converting enzyme (ACE), producing an antihypertensive effect. Additionally, caseinomacropeptide, which is obtained from milk, has demonstrated an effect in platelet aggregation, hence, an antithrombotic activity [90,91].

Moreover, its antimicrobial properties have been proved in corneal wound healing. For example, CAP37, or azurocidin, is considered a bioactive antimicrobial peptide obtained from the granules of neutrophils. CAP37 has been demonstrated to be effective against several bacteria involved in corneal infections including *Pseudomonas Aeruginosa* and *Staphylococcus aureus*. Interestingly, the antimicrobial effects have shown to be mediated by the activation of the protein kinase C signaling pathway in epithelial corneal cells in a mouse model. This activation has been demonstrated to influence corneal epithelial cell migration and accelerate wound closure after topical administration of CAP37 (250–500 ng/mL). In addition, it has been evidenced in mice that CAP37 administered by an intrastromal injection (0.5 μL) results in an increased expression of cytokines such as interleukin 7 (IL-7) or 15 (IL-15) and interferon gamma (IFN-γ), enhancing corneal epithelial recovery [92,93,94].

2. Nutrients: Bioactive Carbohydrates

The bioactive carbohydrates are obtained mainly from plants, including algae, wood plants, dietary fibers, or herbs, from animal tissues such as hyaluronic acid, chondroitin sulfate or heparin, or from microorganisms. Structurally, carbohydrates present a linear (homoglycans) structure such as cellulose, containing the same repeated monosaccharide, or a branched (heteroglycans) structure such as heparin, in which more than one monosaccharide is present. Several therapeutic functions have been attributed to the bioactive carbohydrates, including antioxidant, antimicrobial, antithrombotic, hypoglycemic, or antitumor activities [72,95].

For instance, sulfated polysaccharides obtained from marine algae, including alginate, laminarin and fucoidan, demonstrate great biologic antioxidant activity by inhibiting lipid peroxidation, scavenging nitric oxide, hydroxyl, and superoxide radicals or by inducing glutathione and superoxide dismutase. Moreover, fucoidan has shown to inhibit factor Xa, thrombin, and the intrinsic and extrinsic coagulation cascade, proving its antithrombotic and anticoagulant activities [96,97]. Additionally, the use of fucoidan (400 mL/day) has also been used as an anti-inflammatory agent in cancer patients where it demonstrated a reduction in cytokines including IL-6, TNF-a and IL-1β after its oral administration [98]. Interestingly, a combination of carbohydrates and peptides obtained from mushrooms, known as polysaccharopeptide, has been described to function as immunomodulators, antioxidant, antitumor, or even as a neuroprotective agent [99,100].

3. Nutrients: Fatty Acids

Chemically, a fatty acid is composed of hydrocarbon and carboxylic acid groups from which they can be classified. They can be divided into saturated (single bond) or unsaturated (two or more double bonds) fatty acids [72,101]. The omega-3 fatty acids are unsaturated hydrocarbon chains that are considered essential since they cannot be synthesized in the human body and must be obtained from the diet. Omega-3 fatty acids can be divided according to the length of their fatty acid chains, either short (<6 carbon atoms), including alpha-linoleic acid (ALA), or long (13–22 carbon atoms) chain fatty acids such as docosahexaenoic acid (DHA) and eicosapentaenoic acid (EPA). ALA is mainly obtained from plant-based foods (chia, flaxseed), whereas DHA and EPA are obtained from marine foods such as fish [102]. Among one of the most important functions of omega-3 in the human body is its participation in the reduction in inflammation through competitive inhibition with arachidonic acid (ARA) and important enzymes such as 5-lipoxygenase and cyclooxygenase. To maintain a healthy equilibrium, an optimal ratio of omega-6:omega-3 has been established to be 4:1 [102,103]. Different studies have demonstrated that most patients with Western diets have a ratio that varies from 15:1 to 16.7:1, favoring omega-6 [103]. As a consequence, the excessive amount of omega-6 has shown to increase the synthesis of thromboxane A2, leukotriene B4, interleukin-1ß (IL-1ß), interleukin-6 and tumor necrosis factor (TNF), resulting in the development of common chronic inflammatory pathologies such as cardiovascular disease, cancer, obesity, and autoimmune diseases [104]. Furthermore, including a diet rich in long omega-3 fatty acid may be of potential benefit when treating chronic ocular conditions, such as dry eye disease and age-related macular degeneration [105].

4. Nutrients: Vitamins

Vitamins are chemically heterogenous molecules that have shown an important role in human health when being administered as a nutraceutical. Vitamin A is one of the most well-known micronutrients used as a nutraceutical. The main sources of vitamin A are animal sources, such as salmon, eggs, beef liver, rich beta carotene vegetables including carrots, sweet potato or spinach, or fruits such as mango. The active forms of retinoids include retinol, retinal and retinoic acids, and act through nuclear receptors resulting in gene regulation, and thus exert a crucial role in biologic functions such as reproduction, embryologic development, cellular differentiation, immune health, and vision [106,107,108]. Characteristically, retinoids contain a head-to-tail structure along with four isoprene units. The retinol (vitamin A1) chemical structure includes a ß-ionone ring, an unsaturated isoprenoid side chain (all-trans) and a hydroxide group (-OH). Moreover, the retinal chemical structure is based on four exocyclic double bonds and an attaching group (-CHO). Lastly, retinoic acids, such as all trans-retinoic acid, also present four exocyclic double bonds but with a different attaching group (-COOH) [107,109]. In ocular health, vitamin A has shown to be a key player in maintaining vision. Malnutrition, which is related to vitamin A deficiency, constitutes one of the most common causes of preventable blindness. This reversible deficiency can be prevented by correct nutrition or supplementation, which can restore visual function if it is diagnosed early. On the contrary, a long-term deficiency may result in serious complications, including corneal ulceration and conjunctival keratinization [103].

Another important micronutrient is vitamin B12, also known as cobalamin. This essential micronutrient has been found in animal protein, with meat being the richest source. B12 is one of the most chemically complex vitamins, composed of a tetrapyrrolic combination with four propionamides and three acetamides held within the periphery of the macrocycle and with a central cobalt [110]. Compared to vitamin A, cobalamin is a water-soluble vitamin which has shown a fundamental role in the synthesis of myelin, resulting in peripheral neuropathy, optic nerve atrophy, ineffective erythropoiesis, megaloblastic anemia, and subacute combined degeneration when deficient. Additionally, an important anti-inflammatory function has been attributed to cobalamin due to its ability to modulate NF-kB, which is known to be an activator of an inflammatory pathway [103,111].

Furthermore, vitamin C or ascorbic acid, which is a water-soluble vitamin, is obtained from citrus fruits, strawberries, or broccoli. Structurally, vitamin C is composed of a lactone ring, which has an ethyl diol side chain and two hydroxyl groups. Additionally, it presents four hydroxyl groups which function as proton acceptors or donators, while the ketone and ether groups only function as proton acceptors [112,113]. Due to its unique ability to donate electrons, vitamin C has demonstrated to reduce inflammation and present immunomodulatory, antithrombotic, antiviral, and wound healing properties [114,115]. Interestingly, it has been shown that diabetic patients supplemented with antioxidants vitamin C and E have increased tear production and stability of goblet cells with a consequent decrease in nitric oxide (NO) production. NO is a key molecule in the production of reactive nitrogen species such as peroxynitrite (ONOO-), a potent oxidant that leads to ocular inflammation, though demonstrating its antioxidant abilities [116].

Lastly, vitamin D is considered a fat-soluble vitamin obtained from sunlight exposure, diet and supplementation [117]. Vitamin D presents a unique steroid hormone structure due to the possession of both a seco-B triene structure, which lacks a B-ring, and a complete 25-hydroxycholesterol side chain. This differs from classic steroid hormones, such as progesterone and estradiol, which present a truncated or no side chain [118]. Calcitriol (1,25(OH)2D), the active form of vitamin D, has been established as having an important immunomodulatory function by promoting a lymphocyte shift from Th1 and Th17 to Th2 phenotype, which results in the suppression of pro-inflammatory cytokines (IL-2, interferon-γ, TNF-α) and the expression of anti-inflammatory cytokines (IL-4, IL-13), promoting alternate activation in macrophages [117].

5. Nutrients: Minerals

Along with vitamins, minerals have also proved to be important antioxidants for the human body. One example is selenium, which is found as an amorphous or crystalline form, and mainly presents in a combined form with heavy metals such as mercury, copper and lead. Moreover, selenium can be found as inorganic selenium within various minerals, including selenites and selenates, or as organic selenium as selenoaminoacids, selenopeptides and selenoproteins. The most prevalent organic form within plants entails selenoaminoacids, such as selenomethionine and methylselenocysteine [119]. Selenium can be obtained from meat, eggs, seafood and cereals [103]. The human genome contains 25 selenium-containing proteins whose biologic functions are dependent on the insertion of selenium [103]. The major role of some selenoproteins, such as glutathione peroxidase and thioredoxin reductase, have been demonstrated to act as intracellular antioxidants, thus preventing cell oxidative injury, evidenced in many different pathologies, including ocular diseases. Supplementation with selenium is thought to improve phenylketonuria, atherosclerosis, hypercholesterolemia and type 1 diabetes mellitus, although the role of selenium in type 2 diabetes mellitus is unclear [120].

In addition, zinc is a metallic element in the zinc group of the periodic table present in oxides, sulfides, phosphates, or silicates minerals and is considered the second most prevalent trace element in the human body. It is essential for the function of more than 2800 macromolecules and over 300 enzymes, and is critical for cell proliferation, differentiation, communication, and apoptosis [121,122]. A key function of this element is its role in immunomodulation. Both innate and adaptive immune response are linked to adequate levels of zinc, and the consequent deficiency of this ion leads to inflammation. Other classic features of zinc deficiency include night blindness, anorexia, and weight loss, delayed sexual maturation, testicular atrophy, hypogeusia, alopecia and epidermal hyperkeratinization [121,123,124,125,126]. Moreover, zinc is present in high concentrations in the eye, especially in the retina and choroid, where it has been shown to interact with taurine and vitamin A, modify photoreceptor plasma membranes, serve as an antioxidant, regulate light-rhodopsin reaction and modulate neurotransmission. Lastly, one important group of enzymes known as collagenases depends on zinc availability and may indirectly lead to corneal ulcers when deficient due to abnormal enzyme function [121,124,126].

##### Herbals

Finally, herbals are classified as chemical compound nutraceuticals and have shown different physiologic improvements such as antipyretic, anti-inflammatory, diuretic, or analgesic. Moreover, diseases such as hypertension, cancer, urinary tract infections or COVID-19 have been benefited by the use of these nutraceuticals [41,85,127,128]. One of the most important herbals is *Vaccinium erythrocarpum*, better known as cranberries. Cranberries present rich amounts of polyphenols such as proanthocyanidin, a potent antioxidant. Interestingly, proanthocyanidins have been shown to prevent the recurrence of urinary tract infections (UTIs) by inhibiting the attachment of bacteria and, consequently, the development of chronic kidney disease [129,130,131,132]. Furthermore, the presence of polyphenols in cranberries has proved to be cardioprotective by maintaining blood pressure, lipoproteins, and homocysteine at healthy levels. In addition, they have presented an antioxidant effect through the interference of specific inflammation and oxidative stress pathways such as Nf-Kb and an activation of Nrf2 [129,133]. Importantly, cranberries have also shown a key role in tumorigenesis, either by direct cytotoxicity or inhibition of enzymes involved in cellular proliferation [134].

Two important herbals, willow bark (*Salix nigra*) and lavender (*Lavandula angustifolia*), have demonstrated an impact in arthritis, either collagen-induced or rheumatoid arthritis, exerting anti-inflammatory and antioxidant effects suppression of cytokines, lipid peroxidation and accumulation of free radicals [135,136,137].

Interestingly, Aboutaleb et al. have demonstrated that lavender was implicated in the restoration of antioxidant enzymes (SOD, CAT, GSH) in a rat model of ischemia-reperfusion injury in the kidney [138]. Moreover, psychiatric disorders have also been benefited by the use of lavender, mainly by decreasing anxiety levels after its inhalation [139]. Lastly, several in silico protocols have demonstrated the effect of herbal molecules in diabetic retinopathy and AMD, supporting their role in the limitation of the side effects of ocular drugs used today [140].

#### 4.1.2. Traditional Nutraceuticals: Enzymes

The second primary group of traditional nutraceuticals are the nutraceutical enzymes, which are derived from either plant, microbes, or animals. Enzymes are proteins characteristically composed of amino acid residues (100–2000) which are rearranged into one or various polypeptide chains. These polypeptide chains have been shown to create a tridimensional structure with an active site, where the substrate is intended to bind. This conformational change has been demonstrated to determine their specificity for a single substrate, and thus its catalytic activity [141]. Enzymes prime function has been evidenced to be the enhancement of other protein or fruit preparations with a consequent benefit in the human body [41,142]. Digestive enzymes derived either from plant and bacterial sources, such as pectinase and cellulase, have demonstrated an effect in obesity through the reduction in glutamate pyruvate transaminase, insulin, but most importantly of leptin levels when administered orally with an Ecklonia cava extract in mice models [75]. Moreover, pectinase has been used in addition to a chinese plum concentrate in cell cultures, demonstrating a significant suppression of the proliferation of colorectal cancer cells and angiogenesis of endothelial cells of the umbilical vein [143]. Lastly, hydrolysates of soy protein have shown an immunomodulatory effect when prepared with proteases, including papain and trypsin, presenting an increase in lymphocyte proliferation and in phagocytic activity after its in vitro administration [144].

#### 4.1.3. Traditional Nutraceuticals: Probiotics

The last group of traditional nutraceuticals belongs to the probiotics. As previously explained, probiotics are living microorganisms that can offer beneficial effects to the host if taken in adequate amounts orally. The most common species commercially available are three: *Saccharomyces boulardii, Bifidobacterium, and Lactobacillus* [41,85]. In addition to its antioxidant effect, the strain *S. boulardii* has shown alleviation of gastrointestinal diseases such as pseudomembranous colitis or the adverse effects of helicobacter pylori treatment when administered orally. This strain has proved to directly neutralize enteric bacteria such as *Escherichia coli*, *Salmonella*, *Vibrio cholera*, among others and increase the expression of anti-inflammatory interleukins (IL-1, IL-5, IL-10, IL-12) and decrease the proinflammatory ones including IL-6 and TNF-α [145,146]. Likewise, *Bifidobacterium* has demonstrated to improve gastrointestinal symptoms in celiac disease patients when administered orally (two capsules a day with 2 × 10^9^ colony-forming units per capsule; *B. Longu* 10^9^ colony forming units (CFU)) by increasing serum macrophage inflammatory protein-1B, reducing TNF- a and peripheral CD3+ T lymphocytes, lessening the levels of *Bacteroides fragilis* and IgA in stool or by restoring the microbiota [147,148,149]. *Lactobacilli* administered orally has shown to restore gut microbiota, reducing immune activation as well as gliadin toxicity in celiac disease patients [150]. Likewise, *Lactobacillus casei* has proved to restore the healthy mucosal structure and restore the gut-associated lymphoid tissue (GALT) in celiac disease mouse models [151]. Interestingly, the use of one capsule a day (15 × 10^9^ CFU or 69 mg) of *Bifidobacterium and Lactobacilli* have shown a role in correcting the disruption of the microbiota induced by obesity, where a positive correlation between fat mass, body mass index, and waist circumference, and the quantity of *Bifidobacterium* was demonstrated, resulting in weight loss after being orally administered [152].

#### 4.1.4. Non-Traditional Nutraceuticals: Recombinant Nutraceuticals

Recombinant nutraceuticals refer to all foods that provide energy made with the use of biotechnology. For instance, Malbaša et al. have used kombucha symbiosis, containing different strains of yeast including *Torulopsis* sp., *S. cerevisiae*, *Zygosaccharomyces* sp., *Saccharomyces bisporus*, and *Saccharomycodes ludwigii*, cultured with black or green tea substrates to make the kombucha beverage. The antioxidant properties of the kombucha beverage were demonstrated when added to solutions with hydroxyl or 2,2-diphenyl-1-picrylhydrazyl (DPPH) radicals, mainly by the presence of polyphenols, vitamin C, vitamin B_2_ and citric content in the kombucha beverage [153]. Another example of recombinant nutraceuticals is the lysozyme or muramidase, which have presented bactericidal properties. Yang et al. have demonstrated the nutritional and immunological benefits of it when obtained from transgenic cattle, evidencing the new medical advances when using biotechnology [154].

#### 4.1.5. Non-Traditional Nutraceuticals: Fortified Nutraceuticals

Conversely, food fortified with nutraceuticals are food with nutrients added to the normal product [41]. These nutraceuticals have been studied in the last several years focusing on the amelioration of patients with certain dietary deficiencies that could lead to different diseases [38]. After an extensive review, Cormick et al. stated that calcium fortified products, especially milk, demonstrated to increase the calcium intake and bone mineral density in hip and femoral neck bone, along with an increase in children’s height [155]. Similarly, other authors have demonstrated benefits in the bone mass of patients at risk of fractures after using this fortified formulation [156]. Furthermore, flour fortified with folic acid has proved to prevent neural tube defects, encephalocele or spina bifida when compared to the control groups [157,158]. Finally, the addition of iodine to salt has shown to avoid the development of goiter and non-immune hypothyroidism, which could lead to mental disability, demonstrating its impact on human health [159].

**Figure 1:**
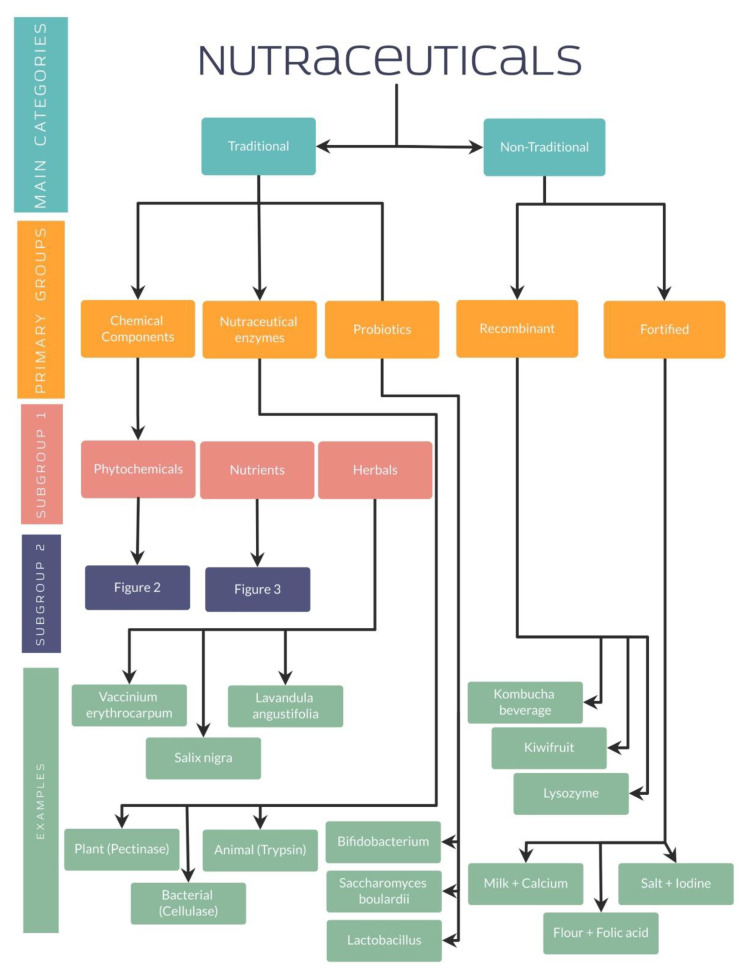
Nutraceutical classification.

**Figure 2:**
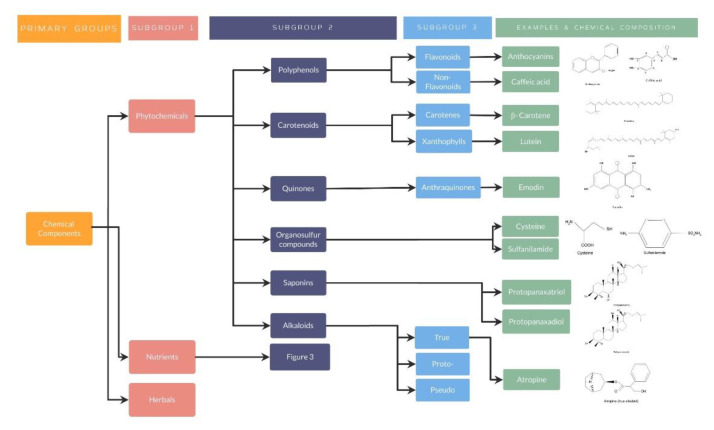
Phytochemicals classification, examples, and chemical composition.

**Figure 3:**
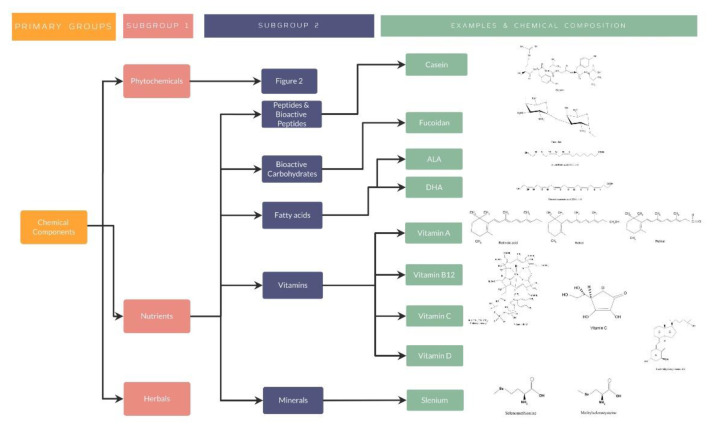
Nutrients classification, examples, and chemical composition.

As well explained, all these studies have evidenced the importance of nutraceuticals in different diseases, especially by its antioxidant effect in different organ systems. For a better understanding, several organization charts (Figure 1) demonstrate a general view of the nutraceutical classification, along with examples and its chemical composition (Figure 2 and Figure 3).

Importantly, it has to be noted that nutraceuticals have also presented a great impact in ophthalmic conditions including presbyopia, DED, glaucoma, or AMD, targeting mainly the oxidative stress involved in their pathophysiology but also in the improvement in the expressed symptoms.

## 5. Use of Nutraceuticals in Ophthalmology

As previously explained, nutraceuticals have demonstrated different benefits in ophthalmic diseases. Since the beginning of the century, genistein and other flavonoids including luteolin and fisetin have shown promising results in ocular pathologies. Joussen et al. have reported a significant inhibition of corneal neovascularization after using a microemulsion and topically administering flavonoids (luteolin: 0.5 mg/mL, genistein: 0.5 mg/mL, fistein: 1 mg/mL) in rabbit models. These hallmark findings have given rise to continuous research on the formulation in ophthalmology [160,161]. Additionally, although prevention was not achieved, it has been demonstrated that genistein (15 mg/kg) administered orally has the ability to hinder the formation of cataracts in rats [162]. Furthermore, a few studies have been carried out regarding non-flavonoid compounds such as caffeic acid, which has been administered intraperitoneally (10 μmol/kg) in rats with induced uveitis by a lipopolysaccharide (LPS) injection or topically (25 μL for six hours every 30 min) in rabbits with paracentesis-induced inflammation of the anterior chamber, and has demonstrated promising results in the attenuation of ocular inflammation, as well as in oxidative stress [163,164]. Similarly, rat models with drug-induced ocular toxicity treated with caffeic acid (10 μmol/kg) administered through an intraperitoneal injection, have shown an increase in the expression of key enzymes responsible for regulating excessive oxidative stress such as superoxide dismutase (SOD), resulting in a significant reduction in overall oxidant status and evidencing the impact of this non-flavonoid compound on ocular stability [165]. Moreover, the role of the intraperitoneal-injected caffeic acid (10 mg/kg) in mitochondrial processes has been evidenced in mice by the reduction in ROS through the uncoupling protein (UCP2), resulting in the preservation of the retinal pigment epithelial cells [166].

In the following paragraphs, the impact of nutraceuticals on the research of the most prevalent ocular disorders (presbyopia, cataract, DED, glaucoma, AMD and diabetic retinopathy) will be described, with special emphasis on the clinical assays.

### 5.1. Presbyopia

Presbyopia is considered a refractive condition in which the gradual loss of accommodation results in the inability to focus on near objects. This is a common age-related eye disorder to which two main causes have been attributed: the progressive weakening of the ciliary muscles and/or a loss of lens elasticity [167,168]. Although investigation of presbyopia regarding nutraceuticals is scarce, a randomized placebo-controlled double blind comparison study was conducted to evaluate the efficacy of various dietary supplements including lutein (10 mg), astaxanthin (4 mg), bilberry extract and black soybean hull extract containing anthocyanin (2.3 mg Cyanidin-3- Glucoside) and docosahexaenoic acid (DHA; 50 mg) on the accommodative ability of the eye. Improvement was observed in eye accommodation and symptoms such as eye strain and blurred vision when looking at objects from near to far after four weeks of ingestion when compared to placebo.

Moreover, anthocyanin has shown relaxant effects, mediated by an endothelin-B receptor and nitric oxide/cyclic GMP pathway on the ciliary muscle and blood vessels, finally improving lens accommodation. On the other hand, astaxanthin has shown to inhibit the NF-kB pathway in the ciliary body, preventing ciliary muscle loss of function and increasing blood flow through the retinal capillaries with a consequent increase in blood flow through the ciliary muscle. Furthermore, DHA may play a role in keeping the ciliary body healthy and enhancing the antioxidant effects of astaxanthin by a mechanism that is yet unknown [169]. A positive accommodation effect given by a mixture of traditional Chinese herbal medicines, containing wolfberry (200 mg), *Cassiae Semen* (200 mg), and *Dendrobium huoshanense* (DD) (40 mg) in a single capsule, was evidenced in a prospective study. It was demonstrated that *Cassiae* seeds enhance the parasympathetic function of the eye, thus improving its accommodative function and the contractility of the ciliary muscle with the consequent relaxation of the zonula fibers. Furthermore, it was shown that *Cassiae Semen* and wolfberry have potent antioxidant properties and may play a role in lowering intraocular pressure (IOP), further relieving presbyopia symptoms [170].

Additionally, Korenfeld et al. have shown a significant amelioration in distance-corrected near vision acuity (DCNVA) bilaterally after the topical administration of lipoic acid (EV06) to presbyopic patients. Similarly, Stein et al. have reported the long-term effects of EV06 after its cessation by evidencing a continuous improvement in near vision [171].

Interestingly, topical pirenoxine (0.005%) has been shown to maintain accommodative amplitude and, consequently, aid in the progression prevention of presbyopia, especially in the fifth decade. In this research, Tsuneyoshi et al. have stated a better accommodative amplitude after its topical administration, demonstrating a difference in diopters in the treatment group of −0.05 ± 0.21 (*p* = 0.59) and −0.16 ± 0.05 in the control group (*p* < 0.01) [10].

### 5.2. Cataract

Cataract is the result of the accumulation of proteins in the lens, which leads to the clouding of it and, finally, to optical deterioration [172]. It is one of the main causes resulting in visual impairment and blindness all over the world, with 47.8% of global blindness attributed to it [172,173,174]. Briefly, the anterior segment of the eye is composed by the cornea, the iris, and the lens. The lens is nourished by the aqueous humor produced by the ciliary processes. It has been found to be composed of cortical and nuclear fiber cells, which contribute to the transparency and execution of its main function: transmitting light to the retina. Any alteration in its transparency due to aging, environment (ultraviolet light), or illness would lead to the disruption of its refractive function [175].

Interestingly, cataracts are classified into four clinical forms, including nuclear, cortical, sub-capsular, and mixed (cortical and nuclear), depending on the anatomic location of the lens clouding. The nuclear type is one of the most common types of cataracts. Characteristically, the central core cells present a normal structural morphology, but there has been demonstrated a loss of sulfhydryl groups of their proteins and an increase in protein disulfides, which results in the aggregation of proteins and disruption of the light transmission [173]. Conversely, in the cortical type, the cortical fiber cells of the lens have shown opacifications in their morphology, tissue liquefaction, and an inability of volume regulation caused by oxidative and osmotic stress. This resulted in cellular swelling and protease activation, leading to insoluble protein aggregation and the scattering of the light [173].

Importantly, the antioxidant function of different nutraceuticals has also been demonstrated, especially in observational studies, to reduce the aggregation of proteins in the lens and, consequently, the incidence and risk of developing cataracts [176]. For instance, the use of multivitamins, vitamin E or vitamin C supplements for more than ten years has resulted in a significant decrease in cortical cataract risk with an odds reduction (OR) of 0.4 (95% CI, 0.2–0.8; *p* = 0.002) [177]. Conversely, although the oral combination of vitamin C (300–600 mg) and E (400 mg) has been demonstrated to protect against lens opacity [178], Vitale et al. have stated that prevention of the lens cataract, especially of the nuclear type, was achieved with only high vitamin E plasma levels (≥12.8 µg/mL) [179]. Moreover, a reduction of 55% of presenting nuclear cataracts after the intake of >90 mg/day of vitamin E for ≥10 years was reported [180,181].

Similarly, several authors have determined the effect of only vitamin C in cataract risk reduction [182]. Valero et al. have described the effectiveness of vitamin C in cataract reduction (OR = 0.66). These authors used the Lens Opacities Classification System (LOCS) II to determine their results after >135 mg of vitamin C was orally taken, which correlated to >49 µmol/L in the blood levels of this antioxidant. Interestingly, in this same research, no other antioxidant including selenium, lycopene, vitamin E, or retinol significantly reduced cataract risk [183]. More recently, another group of authors have shown an inverse association of vitamin C with cataracts, even when classified by cataract types, when taking from 17.6 to 83.9 mg/day of vitamin C orally or presenting plasma levels from 3.1 to 52.5 µmol/mL [184]. Lastly, the intake of ≥363 mg/day of vitamin C proved to be effective in decreasing the risk of cortical cataract by 57% in the Nutrition Vision Project (NVP) [180].

Additionally, vitamin A and carotenoids have proved to be key components of the human lens. For instance, the combination of zeaxanthin and lutein (13.8 ng/g) has demonstrated to be the most important carotenoid of the lens and retina, along with retinol ester (25.6 ng/g), retinol (38.1 ng/g) and, in a very less portion, β-carotene (<0.1 ng/g) [180] Interestingly, several authors have found that higher levels of retinol inversely impact in the risk of nuclear cataracts. Ravindran et al. has shown that even when a weak association was present, retinol did reduce the risk of nuclear (OR = 0.69; 95% CI; 0.56–0.84) and posterior subcapsular (OR = 0.65; 95% CI; 0.5–0.85) cataracts with plasma concentrations between 1.8 and 2.27 µmol/mL [184]. Moreover, the development of cortical cataracts has been limited after orally administering vitamin A for five years with an OR = 0.42 (95% CI; 0.24–0.73) [185].

Although zeaxanthin and lutein, are important elements of the lens, most of the research highlight the lack of influence in the development of cataracts after their oral administration [180,183,184]. However, a reduction in the risk for nuclear cataracts after their individual intake or combined has been demonstrated. For instance, Dherani et al. reported a decrease in nuclear cataracts in an Indian population, presenting plasma zeaxanthin levels of 0.02 µmol/L [186]. On the other side, achieving a blood concentration of 0.18 ± 0.08 of lutein after dietary intake has shown to slow the progression of lens aging correlated by its opacity density [187]. Additionally, Rodríguez et al. have found a reduction in the risk of developing cataracts (*p* < 0.05) in individuals with a daily intake of >3290 µg of lutein [188]. Moreover, the intake of lutein >5.6 mg/day for ≥10 years, determined by food questionnaires, was associated with a lower prevalence of nuclear cataracts after using LOCS III as a measure [181].

Furthermore, it was determined that plasma concentrations of >0.041 µmol/L of zeaxanthin and >0.27 µmol/L of lutein was associated with a reduced nuclear cataract risk after dietary intake [189]. Interestingly, a daily intake of >6 µg/day of zeaxanthin and lutein has demonstrated to decrease the cataract risk by 18% in women [190]. Lastly, Moeller et al. have proved to reduce the prevalence by 32% of nuclear cataract after oral administration of >3 µg of both zeaxanthin and lutein for four years presenting an OR = 0.68 (95% CI; 0.48–0.97) [191].

Interestingly, the positive influence of nutraceuticals in cataracts has also been evidenced in a clinical trial. In this research, a slight impact in cataract progression was demonstrated by using and comparing images from digital retro-illumination with LOCS III and defined by the area of opacity which was measured by an increase in the percentage pixel opaque. During this study, a daily oral antioxidant combination which included vitamin C (750 mg), β-carotene (18 mg), and vitamin E (600 mg) was administered for 3 years [192].

### 5.3. Dry Eye Disease

In addition, DED is a multifactorial chronic disease characterized by ocular symptoms including inflammation, irritation, visual disturbance, burning or foreign body sensation, mainly due to a disequilibrium of the tear film, affecting the ocular surface and adnexa. DED mostly affects older people, mainly over the age of 50 years. Other groups affected include contact lens wearers, postmenopausal women, and patients with autoimmune diseases [103,193,194].

The ocular surface is a functional unit composed by the cornea, conjunctiva, the main lacrimal, accessory and meibomian glands, the apical and basal matrices, the eyelashes and Moll and Zeis glands and the nasolacrimal duct [195]. Briefly, the cornea is composed of a transparent and avascular connective tissue, which functions as the first structural barrier of the eye and further provides three quarters of the refractive power of the eye [196]. Additionally, the conjunctiva, responsible of 95% of the total ocular surface, is composed of a mucous epithelium, endothelial cells, and the stroma. Both the cornea and the conjunctival epithelia produce hydrophilic mucins aiding in the tear stability within the eye surface. Similarly, the main lacrimal gland, along with the accessory glands, comprise the Lacrimal Functional Unit, which secretes water and protective proteins that help to maintain the tear homeostasis. Moreover, the meibomian glands, which are located in the tarsal plates of the eyelids, are glands that play a crucial role in producing the superficial lipid layer of the tear, preventing its evaporation [197]. Lastly, the nasolacrimal epithelial system is considered to control tear outflow and adsorb tear components, thus maintaining adequate tear volume and keeping proper homeostasis between tear secretion and outflow. All of these anatomic components have been demonstrated to be affected by DED and have been considered in the development of new ocular treatments [198,199,200].

There is an estimate of about 16.4 million Americans suffering from DED. Different preclinical and clinical studies have proved that inflammation of the ocular surface along with a chronic immune dysregulation represent a key factor in the pathogenesis of DED. Proinflammatory cytokines, matrix metalloproteinases and chemokines lead to the induction of autoreactive T-helper lymphocytes, which create a vicious cycle of inflammation and damage [103,195]. Furthermore, tear hyperosmolarity has been shown to contribute to DED development, resulting in corneal and conjunctival epithelial cell apoptosis and triggering a concurrent inflammatory cascade that eventually ends in goblet cell loss and tear-film instability [201].

Growing evidence regarding the knowledge of DED pathophysiology have raised the importance of the use of nutraceuticals. Dietary imbalance such as vitamin A or omega-3 fatty acid deficiency has been attributed as an important risk factor for developing DED [103]. Interestingly, it has been demonstrated that omega-3 has anti-inflammatory properties and its supplementation prevents apoptosis of secretory epithelial cells in the meibomian glands [2]. Manifold randomized clinical trials and meta-analyses have demonstrated the efficacy of supplementing omega-3 in DED. Two meta-analyses have confirmed the therapeutic benefit of omega-3 in treating signs and symptoms of DED by showing an improvement in the ocular surface disease index (OSDI), which evaluates DED symptoms through a 12-item questionnaire, tear film-break up time (TBUT), tested after adding fluorescein to the eye and observing the time of rupture of the tear film where the normal value is >10 s, and the Schirmer test, which uses filter paper strips within the conjunctival sac to visualize the wetting of it after 5 min (normal value >10 mm), when compared to placebo [195,202,203,204,205,206].

In addition to the two meta-analyses, it is important to mention that each of the randomized controlled clinical trials used different doses and the source of the omega-3 may differ from one another, potentially affecting the efficacy of each treatment [207,208,209,210,211,212,213,214,215,216]. Even though omega-3 has shown a strong level of evidence in treating dry eye disease, currently, there is no FDA approved formulation nor formal recommendation for the usage of essential fatty acids in the treatment of eye diseases. Optimal dosing of essential fatty acids and duration of treatment constitute potential areas of interest in ophthalmology, especially in ocular surface diseases such as DED [205,213,217].

Furthermore, a study involving 30 male patients with DED showed that short-term supplementation with a fixed dose of 1500 mg of vitamin A improved the quality, but not the quantity, of tears [218]. Recently, two studies have demonstrated that the administration of vitamin B12 via eye drops or intramuscularly also improved dry eye symptoms in patients with severe DED, with or without concurrent neuropathic ocular pain. These findings evidence that DED also has a neurosensory component within its pathophysiology [103].

Additionally, an interesting prospective study was conducted to appraise the effectiveness of a nutraceutical formulation-denominated Brudysec^®^ 1.5 g, which contains omega-3 polyunsaturated fatty acids, vitamins, minerals, and antioxidants, seeking the relief of dry eye disease symptoms. After its oral administration, a significant improvement in the symptoms was shown, along with a reduction in the use of artificial tears and conjunctival hyperemia. Other assessed outcomes included ocular surface damage, tear film stability and tear production, where an improvement was evidenced with an increase in Schirmer test score and tear break up time (TBUT), and, therefore, improving tear secretion and tear film stability [2].

Similarly, Hydroeye^®^, which is another nutraceutical formulation composed of polyunsaturated fatty acids, gamma-linoleic acid (GLA), vitamins and magnesium, has demonstrated an impact in DED pathogenesis. Its efficacy was evidenced in patients with tear dysfunction after its oral administration by a significant reduction in OSDI score, surface asymmetry index, and by the inhibition of dendritic cell maturation, hindering the inflammation [219]. Furthermore, Yamashita et al. have also demonstrated a reduction in DED symptoms with the use of nutraceuticals. After the oral administration of MaquiBright^®^, which was composed of delphinidins and anthocyanins obtained from maqui berry extract, an improvement in the Schirmer’s test, the lacrimal production and eye fatigue alleviation after one month of intake were demonstrated [220,221].

Importantly, DED has also been benefited by the anti-inflammatory and antioxidant properties when using topical nutraceuticals. Kador et al. have demonstrated the maintenance of the tear flow after inducing DED with scopolamine in rat models and administering a topical nutraceutical formulation known as Optixcare EH, which include epigallocatechin gallate (EGCG; 4%), resveratrol (4%), astaxanthin (4%), and ethyl pyruvate (4%) [1,222]. Interestingly, the use of colostrum components, especially topical 2-fucosyl-lactose (0.01, 0.1, and 1%), have been demonstrated to impact TBUT, Schirmer test and tear osmolarity, proving the potential role of nutraceuticals in tear film stability in rabbit models with induced DED [3]. Lastly, VisuEvo^®^, an ophthalmic nutraceutical formulation containing vitamin A, omega-3 (EPA and DHA), and vitamin D3, has also been demonstrated to decrease the inflammation of the ocular surface, increase tear film stability and reestablish tear film composition, stating their role in DED pathophysiology [221,223].

### 5.4. Glaucoma

Another important ophthalmic pathology is glaucoma, which is a disease associated with acute or chronic destruction of the optic nerve with or without concomitant intraocular hypertension. Glaucoma is considered the most common cause of irreversible blindness worldwide with an estimated 57.5 million people affected, mainly of 40 years and older [224]. It can be divided into two main categories: acute and chronic glaucoma. The cornerstone in the pathogenesis is considered the damage of the retinal ganglion cells of the optic nerve related to increased intraocular pressure (IOP). The intraocular pressure is determined by the aqueous humor production by the ciliary body, and excretion through the trabecular meshwork and uveoscleral pathways [225]. Understanding the usefulness and impact of nutraceuticals in glaucoma pathophysiology is an important research topic. More recently, some studies have illustrated the potential benefits of nutraceuticals [4]. For instance, Vetrugno et al. have demonstrated the effects on the IOP after oral administration of forskolin and rutin, where a 10% decrease was evidenced from the first week, independently of their usual therapy [226]. Moreover, Mutolo et al. have used an oral nutraceutical containing *Coleus forskohlii*, carnosine, vitamins B1, B2, B6, homotaurine, folic acid, and magnesium in patients with primary open angle glaucoma (POAG) already in treatment and compensated by IOP-lowering drugs, and have shown a significant decrease in IOP [227]. Nutraceutical formulations, such as BrudyPio 1.5g, based on omega-3 polyunsaturated fatty acids have also shown to have a significant effect on the pathophysiology of glaucoma [228]. Different studies have demonstrated that the use of an omega 3-based nutraceutical improved IOP after 12 months (*p* < 0.01) and increased total plasma antioxidant capacity and the DHA erythrocyte membrane content [228,229]. Additionally, capsules containing concentrated powder of *Crocus sativus* have proved to significantly reduce the IOP in patients >50 years with open angle glaucoma [230]. Similarly, a significant intraocular hypotensive effect was demonstrated by Bonyadi et al. in open angle glaucoma patients treated with timolol and dorzolamide by adding 30 mg/day of saffron, a flower derived spice, as an adjuvant [230]. Ohguro et al. have also described the benefits of using oral nutraceuticals. These authors have shown an enhancement of the blood flow to the optic nerve head (ONH) and, consequently, a delay in the visual field damage progression after the oral administration of black currant anthocyanins for twenty-four months in patients with open angle glaucoma (OAG) [231,232].

Topical nutraceuticals have also been developed particularly to improve retinal function in glaucoma patients. For instance, patients with open angle glaucoma and treated with beta blockers were administered topical citicoline (0.2 g; 3 drops/day) for 4 months. The visual evoked potentials (VEP) and pattern electroretinogram (PERG) were increased, supporting the evidence about the nutraceutical impact on the bioelectrical activity of the visual cortex and the retinal bioelectrical responses [233]. Finally, Parisi et al. also studied the VEP and PERG parameters after topical administration of coenzyme Q10 (100 mg) with vitamin E (500 mg) in open angle glaucoma patients. In the study, an increase in both parameters was shown, resulting in an improvement in the cortical responses and the retinal function [234].

### 5.5. Age Macular Degeneration

Furthermore, age macular degeneration, defined as a degenerative disease of the retina, represents the most common cause of blindness in individuals older than 65 years in developed countries. AMD can be classified into two major forms: dry AMD and wet AMD. Dry AMD is caused by the deposition of drusen, which is yellowish extracellular material, while wet AMD is caused especially by neovascularization. Both can lead to vision impairment and in severe cases to total blindness. Nutraceuticals have shown beneficial effects in AMD. One of the major clinical trials evaluating the impact of nutraceuticals in AMD is the Age-Related Eye Disease Study (AREDS), in which patients were given a formulation containing high-dose vitamins C and E, zinc, and beta carotene. As a result, a statistically significant odds reduction of 0.72 (99% CI, 0.52–0.98; *p* = 0.008) was demonstrated for the development of advanced AMD when compared to the placebo group [235]. The other important clinical trial, the AREDS2, included a formulation where beta-carotene was substituted with lutein/zeaxanthin and demonstrated a hazard ratio of 0.90 (95% CI, 0.82–0.99; *p* = 0.04) between lutein/zeaxanthin vs. no lutein/zeaxanthin formulations of the development of late dry AMD, evidencing more benefits than beta carotene [236].

Importantly, Beatty et al. used Ocuvite, a nutraceutical formulation of lutein, vitamin E, zeaxanthin, copper, vitamin C, and zinc oxide, in patients with early AMD. These authors have reported a better best-corrected visual acuity (BCVA) after its oral administration for 36 months [237]. Similarly, OcuviteDuo, which is composed of vitamin E, vitamin C, cupric oxide, lutein, zinc oxide, EPA, zeaxanthin, and DHA, has demonstrated to influence retinal and visual function in people with early stages of AMD by significantly increasing multifocal electroretinography (mfERG) latency after its oral administration for 60 weeks [238].

Finally, Ma et al. have demonstrated in a meta-analysis the impact of lutein, meso-zeaxanthin and zeaxanthin in the macular pigment optical density (MPOD), which is the functional component of the macula, in dry AMD patients by evidencing a significant increase in it [239,240,241,242]. Furthermore, curcumin, which was used in patients with wet AMD as an oral supplement with anti-vascular endothelial growth factor (VEGF), has shown to improve visual acuity and decrease the total number of intravitreal anti-VEGF injections [243].

### 5.6. Diabetic Retinopathy

Lastly, it is important to outline the influence of nutraceuticals in diabetic retinopathy (DR) which is considered the leading cause of blindness worldwide. The retinal microvasculature has shown to be the main structure affected, resulting in macular edema and ischemia of the tissue. DR can be divided into non proliferative DR (NPDR), characterized by microvascular lesions, such as intraretinal hemorrhages, lipid deposits, microaneurysms or hard exudates, and proliferative DR (PDR), characterized by angiogenesis secondary to vascular endothelial growth factor (VEGF) stimulation [244]. As well as the previously explained benefits nutraceuticals have given to the ocular pathologies, diabetic retinopathy is not an exception. Carotenoids, especially lutein, have shown the greatest focus in the treatment of DR. Moschos et al. have shown the effects of lutein (10 mg), meso-zeaxanthin (10 mg) and zeaxanthin (2 mg) in patients with type 2 diabetes without DR after its oral administration. An improvement in multifocal electroretinography (mfERG) results was shown, expressed as greater density of the retinal response [245]. Moreover, another clinical trial in NPDR patients has shown a minimal amelioration of the contrast sensitivity and visual acuity after taking lutein (10 mg/day) orally [246]. Similarly, Hu et al. have demonstrated in NPDR patients a decreased foveal thickness along with a better visual acuity after zeaxanthin (0.5 mg/day) and lutein (6 mg/day) were orally administered [247]. Furthermore, the use of an oral combination (Diaberet^®^) of vitamin E (30 mg), pycnogenol (50 mg), and coenzyme Q10 (20 mg) has shown a significant reduction in the central macular thickness (CMT) in NPDR patients after six months of intake [248]. On the other hand, no studies have been noticed regarding the role of nutraceuticals in PDR. A comparison of the different nutraceuticals that have been studied in ophthalmology is presented in Table 1.

## 6. Conclusions

Nutraceuticals have shown their safety and efficacy in different clinical assays about ocular pathologies [199]. As previously explained, the use of nutraceuticals, such as Brudysec^®^ 1.5g, BrudyPio 1.5g or AREDS2, has demonstrated significant improvements in eye health, including an amelioration in tear characteristics in patients with DED, a decrease in IOP in patients with glaucoma, or in the prevention of AMD progression in elderly patients [2,228,236]. Importantly, the use of nutraceuticals in ophthalmology, as well as in another medical fields, is as an adjuvant of the primary therapy. Along with the positive outcomes in eye health, an increase in treatment adherence to this therapeutic strategy has been noted, due mainly to its natural origin and lesser adverse effects [251]. Therefore, these compounds have emerged as a promising adjuvant therapeutic approach.

However, despite the potential beneficial results of the nutraceuticals, the high costs of most of them represent a significant disadvantage for use on a regular basis. High cost has been one of the main obstacles leading to failure of treatment adherence in patients with ocular diseases using nutraceuticals. Furthermore, another major setback has relied on the fact that most of the available studies of dietary supplements have no evaluation of plasma basal micronutrient levels or the dietary intake of them. Considering the different variations in every diet, it can potentially comprise a confounder, meaning that the diverse outcomes may be the result of each personal background diet [103]. This fact limits the validity of the nutraceutical studies and, as a consequence, it reduces the clinical use of these compounds.

Lastly, although the nutraceuticals have proved potential therapeutic activity in the ophthalmic pathology, surprisingly, with minimal hazards in humans, there still exists an enormous abyss of lacking information about its actions and adverse effects for different ocular pathologies. For that reason, it is pertinent for health professionals and researchers to continue with preclinical and clinical protocols to develop the science of nutraceuticals with ophthalmic applications to accelerate their implementation as a new therapeutic approach for ocular pathology.

## Figures and Tables

**Table 1:** Comparative table of the use of different nutraceuticals in ophthalmology.

Ocular Condition	Nutraceutical	Type of Study	Description	Outcomes	References
Presbyopia	Combination of DHA, Astaxanthin, Lutein, Cyanidin-3- Glucoside	Clinical Trial	Nutraceutical combination administered orally as two gel capsules/day (Daily dose: 50 mg DHA, 4 mg Astaxanthin, 10 mg Lutein, 2.3 mg Cyanidin-3- Glucoside).	Enhanced near point accommodation and a decrease in eye strain and blurred vision.	[169]
	Ophthalmic Solution: EV06 (lipoic acid choline ester)	Clinical trial	Nutraceutical formulation administered topically one drop in one eye twice/day. (Each with 1.5% lipoic acid choline ester)	Significant amelioration in distance-corrected near vision acuity (DCNVA).	[249]
	Pirenoxine	Clinical trial	Nutraceutical compound administered topically one drop in each eye four/day. (Each with 0.005% pirenoxine)	Maintenance of the accommodative amplitude and aid in the prevention of presbyopia progression.	[10]
Cataract	Vitamin C, β-carotene, and vitamin E	Clinical trial	Nutraceutical formulation administered orally, three capsules/day (Each with vitamin C 200 mg, β-carotene 6 mg, and vitamin E 200 mg).	Slight impact in cataract progression measured by an increase percentage pixel opaque.	[192]
DED	Omega 3	Clinical Trial	Omega 3 fatty acids administered orally, two capsules/day (each with 120 mg DHA, 180 mg of EPA).	Improvement in dry eye symptoms, Nelson grade and TBUT.	[208]
	Omega 3	Clinical Trial	Omega 3 fatty acids administered orally, 8 capsules/day (each with 120 mg DHA, 180 mg of EPA).	Improvement in dry eye symptoms, Nelson grade and TBUT. No changes in Schirmer test values.	[209]
	Omega 3	Clinical Trial	Omega 3 fatty acids administered orally, 4 capsules/day (each with 120 mg DHA, 180 mg of EPA).	Improvement in dry eye symptoms, lens wear comfort, increased TBUT and Nelson scores, improvement in epithelial cell morphology.	[211]
	Omega 3	Clinical Trial	Omega 3 fatty acids administered orally, 4 capsules/day (each with 120 mg DHA, 180 mg of EPA).	Improvement in Meibomian Gland Score, TBUT, Schirmer scores and dry eye symptoms.	[207]
	Omega 3	Clinical Trial	Omega 3 fatty acids administered orally, 2 capsules/day (each with 175 mg DHA, 325 mg EPA).	Improvement in dry eye symptoms, TBUT and Schirmer test values.	[210]
	Omega 3	Clinical Trial	Omega 3 fatty acids administered orally, 4 gel capsules/day (Daily dose: 1680 mg EPA and 560 mg DHA).	Significant improvement in tear osmolarity, OSDI, TBUT and a decrease in Metalloproteinase 9 levels.	[212]
	Omega 3	Clinical Trial	Omega 3 fatty acid administered orally, 5 gelatin capsules/day (each with 400 mg EPA, 200 mg DHA).	No significant differences were observed in DED patients.	[213]
	Omega 3	Clinical Trial	Omega 3 fatty acid administered orally, 15 capsules/day (Daily dose: 1245 mg EPA, 540 mg DHA).	Improvement in dry eye symptoms, TBUT, and rose bengal staining score.	[214]
	Omega 3	Clinical Trial	Omega 3 fatty acid administered orally, 5 capsules/day (Daily dose: 945 mg EPA, 510 mg DHA).	Improvement in tear osmolarity, tear stability, OSDI score, TBUT, ocular bulbar redness. Decrease in IL-17A.	[215]
	Omega 3	Clinical Trial	Omega 3 fatty acid administered orally, 2 capsules/day (each with 180 mg EPA, 120 mg DHA).	Improvement in TBUT, OSDI, Schirmer’s scores.	[250]
	Omega 3	Clinical Trial	Omega 3 fatty acids administered orally, 5 capsules/day (Daily dose: 1000 mg EPA, 500 mg DHA).	Reduction is OSDI, higher corneal total nerve branch density and length on the main fiber, improving tear osmolarity.	[216]
	Vitamin A	Clinical Trial	Vitamin A (Daily dose 1500 mg or 5000 IU) administered orally as a tablet.	Improvement in tear osmolarity and reduction in tear ferning.	[218]
	Brudysec^®^ (Minerals, vitamins, omega-3, and antioxidants)	Clinical Series	Nutraceutical formulation administered orally, 3 capsules/day. Each containing 500 mg w-3 fatty acids: 30 mg DPA, 42.5 mg EPA, 350 mg DHA; 4 mg Vitamin E, 133.3 µg Vitamin A, 26.7 mg Vitamin C, 9.17 µg Selenium, 0.33 mg Magnesium, 1.6 mg Zinc, 0.16 mg Copper; 10.8 mg Tyrosine, 5.83 mg Cysteine, 2 mg Glutathione.	Increase in TBUT and Schirmer test scores, and improvement in symptoms.	[2]
	HydroEye^®^: Polyunsaturated fatty acids, GLA, vitamins and minerals	Clinical Trial	Nutraceutical formulation administered orally as 4 softgels/day each containing: -Omega 3 Fatty acids: 196 mg ALA, 126 mg EPA, 99 mg DHA, -Omega 6 fatty acids: 710 mg LA, <30 mg ARA, 240 mg GLA. -Vitamins: 2180 IU vitamin A, 12.8 mg vitamin B6, 262 mg vitamin C, 13.7 mg vitamin E. 8 mg -Magnesium 40 mg	Improvement in OSDI, surface asymmetry index, inhibited dendritic cell maturation. No effect in tear production, TBUT.	[219]
	MaquiBright^®^: Anthocyanins and delphinidins	Clinical trial	Nutraceutical formulation administered orally as 1 capsule/day each containing: 120 mg of Dextrin, 4 mg delphinidin-3,5-O-diglucoside, 21 mg anthocyanins, and 15 mg delphinidins	Eye dryness relief, improvement in Schirmer test and eye fatigue alleviation.	[220]
	Topical nutraceutical: Colostrum (2-fucosyl-lactose)	Animal model: rabbit	2-fucosyl-lactose was administered topically at 3 different doses (0.01%, 0.1% and 1%).	TBUT, Schirmer test and tear osmolarity were improved.	[3]
	Optixcare EH: Topical nutraceutical	Animal model: rat	Nutraceutical formulation administered topically, 2 drops/day. Each drop containing EGCG (4%), resveratrol (4%), astaxanthin (4%), and ethyl pyruvate (4%).	Increased maintenance of tear flow.	[1]
	VisuEvo^®^: vitamin A, omega-3, and vitamin D3.	Clinical trial	Nutraceutical formulation administered topically, one drop/three times a day. Each drop with vitamin A, omega-3 (DHA and EPA), and vitamin D3	Improved ocular surface inflammation, restoration of tear film stability and composition.	[223]
Glaucoma	Kronek^®^: Forskolin, Rutin, Vitamin B1 and B2	Clinical trial	Nutraceutical formulation administered orally, 2 tablets/day (each with 0.7 mg vitamin B1, 0.8 vitamin B2, 15 mg Forskolin, 200 mg Rutin).	Reduction in intraocular pressure.	[226]
	Gangliolife^®^: Carnosine, Forskolin, folic acid, homotaurine, vitamins and magnesium	Clinical Trial	Combined nutraceutical administered orally, 2 tablets/day (each with 50 mg Carnosine, 15 mg Forskolin, 0.2 mg Folic acid, 100 mg, Homotaurine, 1.4 mg vitamin B6, 1.4 mg vitamin B2, 1.1 mg vitamin B1, 150 mg Magnesium).	Increase in foveal sensitivity, better PERG and a reduction in IOP.	[227]
	BrudyPio: Vitamins, minerals, fatty acids, and antioxidants	Clinical Trial	Nutraceutical formulation, administered orally, 3 capsules/day. Each containing: 30 mg DPA, 42.5 mg EPA, 350 mg DHA; 4 mg Vitamin E, 133.3 µg Vitamin A, 26.7 mg Vitamin C, 0.36 mg Vitamin B1, 0.46 mg Vitamin B2, 5.33 mg Vitamin B3, 0.46 mg Vitamin B6, 66.7 µg Vitamin B9, 0.83 µg Vitamin B12, 18.3 µg Selenium, 0.66 mg Manganese, 3.33 mg Zinc, 0.33 mg Copper; 0.33 mg Zeaxanthin, 3.33 mg Lutein, 2 mg Glutathione, 2 mg Coenzyme Q10, 2 mg Lycopene, 67 µg Oleuropein, 5 mg Anthocyanins.	Decrease in IOP and proinflammatory cytokines.	[228]
	Saffron (Crocus Sativus)	Clinical Trial	Saffron administered orally, 1 capsule/day (30 mg).	Reduction of IOP after three weeks of administration.	[230]
	Black currant anthocyanins (BCACs)	Clinical Trial	BCACs administered orally, 2 capsules/day (50 mg/day)	Enhancement of the blood flow to the ONH and delay in the visual field damage progression.	[232]
	OMK1^®^: HA, citicoline and benzalkonium chloride.	Clinical trial	OMK1 administered topically, 3 drops/day (each containing 0.02 g HA, 0.2 g citicoline, 0.001 g benzalkonium chloride).	Significant increase in PERG and shortened VEP.	[233]
	Coqun^®^: Coenzyme Q10 and vitamin E	Clinical trial	Nutraceutical formulation administered topically, 2 drops/day (each with 100 mg Coenzyme Q10 with 500 mg vitamin E).	Beneficial effect on the retinal function (PERG improvement) and enhancement of visual cortical responses (VEP improvement).	[234]
AMD	AREDS: Beta-carotene, Vitamin C and E, and Zinc	Clinical Trial	Nutraceutical formulation administered orally, 4 tablets/day (each with 15 mg Beta-carotene, 500 mg Vitamin C, 400 IU Vitamin E, 80 mg Zinc).	Decrease progression to advanced disease.	[235]
	AREDS 2: Lutein/zeaxanthin, Vitamin C and E, and Zinc	Clinical Trial	Nutraceutical formulation administered orally, 4 pills/day (each with 10 mg/2 mg Lutein/zeaxanthin, 500 mg Vitamin C, 400 IU Vitamin E, 25 mg Zinc).	Decrease progression to advanced disease, mainly neovascular AMD.	[236]
	Ocuvite: lutein, vitamin E, zeaxanthin, copper, vitamin C, and zinc oxide	Clinical Trial	Nutraceutical formulation administered orally, 1 pill/twice daily (Each with lutein 12 mg, vitamin E 15 mg, zeaxanthin 0.6 mg, copper 0.4 mg, vitamin C 150 mg, zinc oxide 20 mg)	Significant difference in the best-corrected visual acuity (BCVA).	[237]
	Ocuvite Duo: vitamin E, vitamin C, cupric oxide, lutein, zinc oxide, EPA, zeaxanthin, and DHA	Clinical Trial	Nutraceutical formulation administered orally, 1 pill/day (Each with vitamin E 15 mg, vitamin C 150 mg, cupric oxide 400 μg, lutein 12 mg, zinc oxide 20 mg, EPA 240 mg, zeaxanthin 0.6 mg, and DHA 840 mg)	Significant increase in multifocal electroretinography (mfERG) latency.	[238]
	Lutamax DUO: Lutein (L)	Clinical Trial	Nutraceutical formulation administered orally, 1 tablet/day (20 mg L for 3 months, then 10 mg L for another 3 months).	Increased MPOD, no effect on macular function or visual activity.	[240]
	Lutein (L), zeaxanthin (Z)	Clinical Trial	Nutraceutical formulation administered orally, 1 pill/day (Each with 8 mg L, 20 mg Z, 190 mg mixed fatty acids; or pure 20 mg Z).	Improvement in the temporal contrast sensitivity function (tCSF) and MPOD. Increased the visual processing speed.	[241]
	Lutein (L), Zeaxanthin (Z), DHA	Clinical Trial	Nutraceutical formulation administered orally, 2 tablets/day (Daily dose: 0.6 mg Z, 12 mg L, 280 mg DHA).	Improvement in MPOD.	[242]
	Curcumin	Clinical Trial	Curcumin administered orally/day.	Improve visual acuity and decrease the total number of intravitreal anti-VEGF injections.	[243]
Diabetic retinopathy (non-proliferative)	Carotenoids	Clinical trial	Nutraceutical formulation administered orally, 1 capsule/day (Daily dose: Lutein 10 mg, meso-zeaxanthin 10 mg, zeaxanthin 2 mg).	Increase in foveal thickness and improvement in mfERG.	[245]
	Lutein	Clinical trial	Lutein (10 mg/day) administered orally, 1 capsule/day.	Improvement in contrast sensitivity and visual acuity.	[246]
	Lutein and Zeaxanthin	Clinical trial	Lutein (6 mg) and zeaxanthin (0.5 mg) administered orally/day.	Improvement in visual acuity and decreased foveal thickness.	[247]
	Diaberet^®^: vitamin E, pycnogenol, and coenzyme Q10	Clinical trial	Nutraceutical formulation administered orally 1 tablet/day. (Daily dose: vitamin E 30 mg, pycnogenol 50 mg, and coenzyme Q10 20 mg).	Improvement in central macular thickness.	[248]

DHA: docosahexaenoic acid; DCNVA: distance-corrected near vision acuity; DED: dry eye disease; EPA: eicosapentaenoic acid; TBUT: tear film-break up time; OSDI: ocular surface disease index; DPA: docosapentaenoic acid; GLA: gamma-linolenic acid; ALA: alpha-linolenic acid; LA: linoleic acid; ARA: arachidonic acid; EGCG: epigallocatechin gallate; PERG: pattern electroretinogram; IOP: intraocular pressure; HA: hyaluronic acid; BCACs: black currant anthocyanins; ONH: optic nerve head; VEP: visual evoked potentials; AMD: age macular degeneration; tCSF: temporal contrast sensitivity function; BCVA: best-corrected visual acuity; mfERG: multifocal electroretinography; MPOD: macular pigment optical density; VEGF: vascular endothelial growth factor.

## Data Availability

The data presented in this study are available on request from the corresponding author.

## References

[1] Kador P.F. (2017). Topical applied nutraceutical antioxidant formulation reduces ocular oxidative stress. Funct. Foods Health Dis..

[2] Gatell-Tortajada J. (2016). Oral supplementation with a nutraceutical formulation containing omega-3 fatty acids, vitamins, minerals, and antioxidants in a large series of patients with dry eye symptoms: Results of a prospective study. Clin. Interv. Aging.

[3] Bucolo C., Musumeci M., Salomone S., Romano G.L., Leggio G.M., Gagliano C., Reibaldi M., Avitabile T., Uva M.G., Musumeci S. (2015). Effects of topical fucosyl-lactose, a milk oligosaccharide, on dry eye model: An example of nutraceutical candidate. Front. Pharmacol..

[4] Scuteri D., Rombolà L., Watanabe C., Sakurada S., Corasaniti M.T., Bagetta G., Tonin P., Russo R., Nucci C., Morrone L.A. (2020). Impact of nutraceuticals on glaucoma: A systematic review. Prog. Brain Res..

[5] López-Varela S., González-Gross M., Marcos A. (2002). Functional foods and the immune system: A review. Eur. J. Clin. Nutr..

[6] Asgary S., Rastqar A., Keshvari M. (2018). Functional Food and Cardiovascular Disease Prevention and Treatment: A Review. J. Am. Coll. Nutr..

[7] González-Sarrías A., Larrosa M., García-Conesa M.T., Tomás-Barberán F.A., Espín J.C. (2013). Nutraceuticals for older people: Facts, fictions and gaps in knowledge. Maturitas.

[8] Ronis M.J.J., Pedersen K.B., Watt J. (2018). Adverse Effects of Nutraceuticals and Dietary Supplements. Annu. Rev. Pharmacol. Toxicol..

[9] Nagashima H., Sasaki N., Amano S., Nakamura S., Hayano M., Tsubota K. (2021). Oral administration of resveratrol or lactic acid bacterium improves lens elasticity. Sci. Rep..

[10] Tsuneyoshi Y., Higuchi A., Negishi K., Tsubota K. (2017). Suppression of presbyopia progression with pirenoxine eye drops: Experiments on rats and non-blinded, randomized clinical trial of efficacy. Sci. Rep..

[11] Ishikawa Y., Hashizume K., Kishimoto S., Tezuka Y., Nishigori H., Yamamoto N., Kondo Y., Maruyama N., Ishigami A., Kurosaka D. (2012). Effect of vitamin C depletion on UVR-B induced cataract in SMP30/GNL knockout mice. Exp. Eye Res..

[12] Blondin J., Baragi V., Schwartz E., Sadowski J.A., Taylor A. (1986). Delay of UV-induced eye lens protein damage in guinea pigs by dietary ascorbate. J. Free Radic. Biol. Med..

[13] Ozkaya D., Naziroglu M., Armagan A., Demirel A., Koroglu B.K., Colakoglu N., Kukner A., Sonmez T.T. (2011). Dietary vitamin C and E modulates oxidative stress induced-kidney and lens injury in diabetic aged male rats through modulating glucose homeostasis and antioxidant systems. Cell Biochem. Funct..

[14] Chen M., Hu D.N., Pan Z., Lu C.W., Xue C.Y., Aass I. (2010). Curcumin protects against hyperosmoticity-induced IL-1β elevation in human corneal epithelial cell via MAPK pathways. Exp. Eye Res..

[15] Park B., Lee I.S., Hyun S.W., Jo K., Lee T.G., Kim J.S., Kim C.S. (2018). The Protective Effect of Polygonum cuspidatum (PCE) Aqueous Extract in a Dry Eye Model. Nutrients.

[16] Chien K.J., Horng C.T., Huang Y.S., Hsieh Y.H., Wang C.J., Yang J.S., Lu C.C., Chen F.A. (2018). Effects of Lycium barbarum (goji berry) on dry eye disease in rats. Mol. Med. Rep..

[17] Li L., Jin R., Li Y., Yoon H.S., Yoon H.J., Yoon K.C. (2021). Effects of eye drops containing a mixture of 3% diquafosol sodium and tocopherol acetate (vitamin E) on the ocular surface of murine dry eye. Cutan. Ocul. Toxicol..

[18] Kamalden T.A., Ji D., Fawcett R.J., Osborne N.N. (2011). Genistein blunts the negative effect of ischaemia to the retina caused by an elevation of intraocular pressure. Ophthalmic Res..

[19] Davis B.M., Pahlitzsch M., Guo L., Balendra S., Shah P., Ravindran N., Malaguarnera G., Sisa C., Shamsher E., Hamze H. (2018). Topical Curcumin Nanocarriers are Neuroprotective in Eye Disease. Sci. Rep..

[20] Chichili G.R., Nohr D., Frank J., Flaccus A., Fraser P.D., Enfissi E.M.A., Biesalski H.K. (2006). Protective effects of tomato extract with elevated Β-carotene levels on oxidative stress in ARPE-19 cells. Br. J. Nutr..

[21] Bhatt P., Fnu G., Bhatia D., Shahid A., Sutariya V. (2020). Nanodelivery of Resveratrol-Loaded PLGA Nanoparticles for Age-Related Macular Degeneration. AAPS PharmSciTech 2020.

[22] Sasaki M., Ozawa Y., Kurihara T., Kubota S., Yuki K., Noda K., Kobayashi S., Ishida S., Tsubota K. (2010). Neurodegenerative influence of oxidative stress in the retina of a murine model of diabetes. Diabetologia.

[23] Kowluru R.A., Zhong Q., Santos J.M., Thandampallayam M., Putt D., Gierhart D.L. (2014). Beneficial effects of the nutritional supplements on the development of diabetic retinopathy. Nutr. Metab..

[24] Singh P., Tripathi M.K., Yasir M., Khare R., Tripathi M.K., Shrivastava R. (2020). Potential Inhibitors for SARS-CoV-2 and Functional Food Components as Nutritional Supplement for COVID-19: A Review. Plant Foods Hum. Nutr..

[25] Davidson M.H., Maki K.C., Dicklin M.R., Feinstein S.B., Witchger M.S., Bell M., McGuire D.K., Provost J.C., Liker H., Aviram M. (2009). Effects of Consumption of Pomegranate Juice on Carotid Intima-Media Thickness in Men and Women at Moderate Risk for Coronary Heart Disease. Am. J. Cardiol..

[26] Lionetti V., Tuana B.S., Casieri V., Parikh M., Pierce G.N. (2019). Importance of functional food compounds in cardioprotection through action on the epigenome. Eur. Heart J..

[27] Alkhatib A., Tsang C., Tiss A., Bahorun T., Arefanian H., Barake R., Khadir A., Tuomilehto J. (2017). Functional Foods and Lifestyle Approaches for Diabetes Prevention and Management. Nutrients.

[28] Al Alawi R., Alhamdani M.S.S., Hoheisel J.D., Baqi Y. (2020). Antifibrotic and tumor microenvironment modulating effect of date palm fruit (Phoenix dactylifera L.) extracts in pancreatic cancer. Biomed. Pharmacother..

[29] Surh Y.J. (2003). Cancer chemoprevention with dietary phytochemicals. Nat. Rev. Cancer.

[30] Coleman A.L., Stone K.L., Kodjebacheva G., Yu F., Pedula K.L., Ensrud K.E., Cauley J.A., Hochberg M.C., Topouzis F., Badala F. (2008). Glaucoma Risk and the Consumption of Fruits and Vegetables Among Older Women in the Study of Osteoporotic Fractures. Am. J. Ophthalmol..

[31] Al Owaifeer A.M., Al Taisan A.A. (2018). The Role of Diet in Glaucoma: A Review of the Current Evidence. Ophthalmol. Ther..

[32] Ameratunga R., Woon S.T. (2010). Anaphylaxis to hyperallergenic functional foods. Allergy Asthma Clin. Immunol..

[33] Ameratunga R., Crooks C., Simmons G., Woon S.T. (2015). Health Risks and Adverse Reactions to Functional Foods. Crit. Rev. Food Sci. Nutr..

[34] Fernando S.L., Clarke L.R. (2009). Salicylate intolerance: A masquerader of multiple adverse drug reactions. Case Rep..

[35] Brazier N.C., Levine M.A.H. (2003). Drug-herb interaction among commonly used conventional medicines: A compendium for health care professionals. Am. J. Ther..

[36] Low Dog T., Markham M.J. (2013). Dietary Supplements and Hemostasis. Consult. Hemost. Thromb..

[37] Santini A., Cammarata S.M., Capone G., Ianaro A., Tenore G.C., Pani L., Novellino E. (2018). Nutraceuticals: Opening the debate for a regulatory framework. Br. J. Clin. Pharmacol..

[38] Aronson J.K., Aronson J.K. (2017). Defining ‘nutraceuticals’: Neither nutritious nor pharmaceutical. Br. J. Clin. Pharmacol..

[39] Rojas Jiménez S., Sebastián J., Valle L., Ocampo A.U., Correa Pérez S., Perilla Hernández N., Sebastián J., Cárdenas M. (2015). Consumo de nutracéuticos, una alternativa en la prevención de las enfermedades crónicas no transmisibles. Biosalud.

[40] Nwosu O.K., Ubaoji K.I. (2020). Nutraceuticals: History, Classification and Market Demand. Functional Foods and Nutraceuticals.

[41] Alamgir A.N.M. (2018). Vitamins, Nutraceuticals, Food Additives, Enzymes, Anesthetic Aids, and Cosmetics. Progress in Drug Research.

[42] Calis Z., Mogulkoc R., Baltaci A.K. (2020). The Roles of Flavonols/Flavonoids in Neurodegeneration and Neuroinflammation|Bentham Science. Mini Rev. Med. Chem..

[43] Alamgir A.N.M. (2017). Classification of Drugs, Nutraceuticals, Functional Food, and Cosmeceuticals; Proteins, Peptides, and Enzymes as Drugs. Progress in Drug Research.

[44] Singla R.K., Dubey A.K., Garg A., Sharma R.K., Fiorino M., Ameen S.M., Haddad M.A., Al-Hiary M. (2019). Natural polyphenols: Chemical classification, definition of classes, subcategories, and structures. J. AOAC Int..

[45] Devi S., Kumar V., Singh S.K., Dubey A.K., Kim J.-J. (2021). Flavonoids: Potential Candidates for the Treatment of Neurodegenerative Disorders. Biomedicines.

[46] Abotaleb M., Samuel S.M., Varghese E., Varghese S., Kubatka P., Liskova A., Busselberg D. (2018). Flavonoids in Cancer and Apoptosis. Cancers.

[47] Zhang H.W., Hu J.J., Fu R.Q., Liu X., Zhang Y.H., Li J., Liu L., Li Y.N., Deng Q., Luo Q.S. (2018). Flavonoids inhibit cell proliferation and induce apoptosis and autophagy through downregulation of PI3Kgamma mediated PI3K/AKT/mTOR/p70S6K/ULK signaling pathway in human breast cancer cells. Sci. Rep..

[48] Guan L.P., Liu B.Y. (2016). Antidepressant-like effects and mechanisms of flavonoids and related analogues. Eur. J. Med. Chem..

[49] Nabavi S.M., Daglia M., Braidy N., Nabavi S.F. (2017). Natural products, micronutrients, and nutraceuticals for the treatment of depression: A short review. Nutr. Neurosci..

[50] Verma S., Singh A., Mishra A. (2013). Gallic acid: Molecular rival of cancer. Environ. Toxicol. Pharmacol..

[51] Liu G., Zhang B.-f., Hu Q., Liu X.-p., Chen J. (2020). Syringic acid mitigates myocardial ischemia reperfusion injury by activating the PI3K/Akt/GSK-3β signaling pathway. Biochem. Biophys. Res. Commun..

[52] Salau V.F., Erukainure O.L., Islam M.S. (2021). Caffeic Acid Protects against Iron-Induced Cardiotoxicity by Suppressing Angiotensin-Converting Enzyme Activity and Modulating Lipid Spectrum, Gluconeogenesis and Nucleotide Hydrolyzing Enzyme Activities. Biol. Trace Elem. Res..

[53] Zhang Y., Kong D., Han H., Cao Y., Zhu H., Cui G. (2022). Caffeic acid phenethyl ester protects against doxorubicin-induced cardiotoxicity and increases chemotherapeutic efficacy by regulating the unfolded protein response. Food Chem. Toxicol..

[54] Neto-Neves E.M., da Silva Maia Bezerra Filho C., Dejani N.N., de Sousa D.P. (2021). Ferulic Acid and Cardiovascular Health: Therapeutic and Preventive Potential. Mini Rev. Med. Chem..

[55] Li D., Rui Y.-x., Guo S.-d., Luan F., Liu R., Zeng N. (2021). Ferulic acid: A review of its pharmacology, pharmacokinetics and derivatives. Life Sci..

[56] Li S., Huang Q., Zhang L., Qiao X., Zhang Y., Tang F., Li Z. (2019). Effect of CAPE-pNO2 against type 2 diabetes mellitus via the AMPK/GLUT4/ GSK3β/PPARα pathway in HFD/STZ-induced diabetic mice. Eur. J. Pharmacol..

[57] Zduńska K., Dana A., Kolodziejczak A., Rotsztejn H. (2018). Antioxidant properties of ferulic acid and its possible application. Ski. Pharmacol. Physiol..

[58] Wang P., Ye X.-l., Liu R., Chen H.-l., Liang X., Li W.-l., Zhang X.-d., Qin X.-j., Bai H., Zhang W. (2013). Mechanism of acute lung injury due to phosgene exposition and its protection by cafeic acid phenethyl ester in the rat. Exp. Toxicol. Pathol..

[59] Mu M., Zuo S., Wu R.M., Deng K.S., Lu S., Zhu J.J., Zou G.L., Yang J., Cheng M.L., Zhao X.K. (2018). Ferulic acid attenuates liver fibrosis and hepatic stellate cell activation via inhibition of TGF- β/Smad signaling pathway. Drug Des. Dev. Ther..

[60] Ellison S.L. (2016). Carotenoids: Physiology. Encycl. Food Health.

[61] Papas A.M. (2021). Vitamin E TPGS and its applications in nutraceuticals. Nutraceuticals.

[62] Harrison E.H., Curley R.W. (2016). Carotenoids and Retinoids: Nomenclature, Chemistry, and Analysis. Subcell. Biochem..

[63] Toti E., Chen C.-Y.O., Palmery M., Villaño Valencia D., Peluso I. (2018). Non-Provitamin A and Provitamin A Carotenoids as Immunomodulators: Recommended Dietary Allowance, Therapeutic Index, or Personalized Nutrition?. Oxidative Med. Cell. Longev..

[64] Fazal Y., Fatima S.N., Shahid S.M., Mahboob T. (2016). Nephroprotective effects of b-carotene on ACE gene expression, oxidative stress and antioxidant status in thioacetamide induced renal toxicity in rats. Pak. J. Pharm. Sci..

[65] Kavalappa Y.P., Gopal S.S., Ponesakki G. (2021). Lutein inhibits breast cancer cell growth by suppressing antioxidant and cell survival signals and induces apoptosis. J. Cell. Physiol..

[66] Chien S.-C., Wu Y.-C., Chen Z.-W., Yang W.-C. (2015). Naturally Occurring Anthraquinones: Chemistry and Therapeutic Potential in Autoimmune Diabetes. Evid.-Based Complement. Altern. Med..

[67] Malik E.M., Müller C.E. (2016). Anthraquinones As Pharmacological Tools and Drugs. Med. Res. Rev..

[68] Li Y., Jiang J.G. (2018). Health functions and structure–activity relationships of natural anthraquinones from plants. Food Funct..

[69] Abderrahmane B., Noureddine C., Meriem D., Haythem A.S., Arrar L., Mohammad S.M. (2011). Free radical scanvenging and antioxidant effects of some anthraquinone derivatives. Med. Chem..

[70] Goncharov N.V., Belinskaia D.A., Ukolov A.I., Jenkins R.O., Avdonin P.V. (2021). Organosulfur compounds as nutraceuticals. Nutraceuticals.

[71] Ruhee R.T., Roberts L.A., Ma S., Suzuki K. (2020). Organosulfur Compounds: A Review of Their Anti-inflammatory Effects in Human Health. Front. Nutr..

[72] Chukwuebuka E., Genevieve T. (2020). Functional Foods and Nutraceuticals.

[73] Petropoulos S., Di Gioia F., Ntatsi G. (2017). Vegetable Organosulfur Compounds and their Health Promoting Effects. Curr. Pharm. Des..

[74] Khubber S., Hashemifesharaki R., Mohammadi M., Gharibzahedi S.M.T. (2020). Garlic (*Allium sativum* L.): A potential unique therapeutic food rich in organosulfur and flavonoid compounds to fight with COVID-19. Nutr. J..

[75] Kim I.H., Choi J.W., Lee M.K., Kwon C.J., Nam T.J. (2018). Anti-obesity effects of pectinase and cellulase enzyme-treated Ecklonia cava extract in high-fat diet-fed C57BL/6N mice. Int. J. Mol. Med..

[76] Moriarty R., Naithani R., Surve B. (2007). Organosulfur compounds in cancer chemoprevention. Mini Rev. Med. Chem..

[77] Percival S.S. (2016). Aged Garlic Extract Modifies Human Immunity. J. Nutr..

[78] Nantz M.P., Rowe C.A., Muller C.E., Creasy R.A., Stanilka J.M., Percival S.S. (2012). Supplementation with aged garlic extract improves both NK and γδ-T cell function and reduces the severity of cold and flu symptoms: A randomized, double-blind, placebo-controlled nutrition intervention. Clin. Nutr..

[79] Rajagopal K., Byran G., Jupudi S., Vadivelan R. (2022). Activity of phytochemical constituents of black pepper, ginger, and garlic against coronavirus (COVID-19): An *in silico* approach. Int. J. Health Allied Sci..

[80] Pandey P., Khan F., Kumar A., Srivastava A., Jha N.K. (2020). Screening of potent inhibitors against 2019 novel coronavirus (COVID-19) from alliumsativum and allium cepa: An in silico approach. Biointerface Res. Appl. Chem..

[81] Marrelli M., Conforti F., Araniti F., Statti G.A. (2016). Effects of Saponins on Lipid Metabolism: A Review of Potential Health Benefits in the Treatment of Obesity. Molecules.

[82] Shi J., Arunasalam K., Yeung D., Kakuda Y., Mittal G., Jiang Y. (2004). Saponins from Edible Legumes: Chemistry, Processing, and Health Benefits. J. Med. Food.

[83] Kwon D.Y., Kim Y.S., Ryu S.Y., Choi Y.H., Cha M.R., Yang H.J., Park S. (2012). Platyconic acid, a saponin from Platycodi radix, improves glucose homeostasis by enhancing insulin sensitivity in vitro and in vivo. Eur. J. Nutr..

[84] Casciaro B., Mangiardi L., Cappiello F., Romeo I., Loffredo M.R., Iazzetti A., Calcaterra A., Goggiamani A., Ghirga F., Mangoni M.L. (2020). Naturally-Occurring Alkaloids of Plant Origin as Potential Antimicrobials against Antibiotic-Resistant Infections. Molecules.

[85] Sachdeva V., Roy A., Bharadvaja N. (2020). Current Prospects of Nutraceuticals: A Review. Curr. Pharm. Biotechnol..

[86] Kim K.H., Noh H.J., Choi S.U., Lee K.R. (2012). Isohericenone, a new cytotoxic isoindolinone alkaloid from Hericium erinaceum. J. Antibiot..

[87] Arpha K., Phosri C., Suwannasai N., Mongkolthanaruk W., Sodngam S. (2012). Astraodoric acids A-D: New lanostane triterpenes from edible mushroom astraeus odoratus and their anti-mycobacterium tuberculosis H37Ra and cytotoxic activity. J. Agric. Food Chem..

[88] Chakrabarti S., Guha S., Majumder K. (2018). Food-Derived Bioactive Peptides in Human Health: Challenges and Opportunities. Nutrients.

[89] Sánchez A., Vázquez A. (2017). Bioactive peptides: A review. Food Qual. Saf..

[90] Mohanty D.P., Mohapatra S., Misra S., Sahu P.S. (2016). Milk derived bioactive peptides and their impact on human health—A review. Saudi J. Biol. Sci..

[91] Park Y.W., Nam M.S. (2015). Bioactive Peptides in Milk and Dairy Products: A Review. Food Sci. Anim. Resour..

[92] Griffith G.L., Kasus-Jacobi A., Pereira H.A. (2017). Bioactive Antimicrobial Peptides as Therapeutics for Corneal Wounds and Infections. Adv. Wound Care.

[93] Griffith G.L., Kasus-Jacobi A., Lerner M.R., Anne Pereira H. (2014). Corneal Wound Healing, a Newly Identified Function of CAP37, Is Mediated by Protein Kinase C Delta (PKCδ). Investig. Ophthalmol. Vis. Sci..

[94] Lee T.D., Gonzalez M.L., Kumar P., Chary-Reddy S., Grammas P., Pereira H.A. (2002). CAP37, a novel inflammatory mediator: Its expression in endothelial cells and localization to atherosclerotic lesions. Am. J. Pathol..

[95] Liu J., Willför S., Xu C. (2015). A review of bioactive plant polysaccharides: Biological activities, functionalization, and biomedical applications. Bioact. Carbohydr. Diet. Fibre.

[96] Wijesekara I., Pangestuti R., Kim S.K. (2011). Biological activities and potential health benefits of sulfated polysaccharides derived from marine algae. Carbohydr. Polym..

[97] Wang Y., Xing M., Cao Q., Ji A., Liang H., Song S. (2019). Biological Activities of Fucoidan and the Factors Mediating Its Therapeutic Effects: A Review of Recent Studies. Mar. Drugs.

[98] Takahashi H., Kawaguchi M., Kitamura K., Narumiya S., Kawamura M., Tengan I., Nishimoto S., Hanamure Y., Majima Y., Tsubura S. (2018). An Exploratory Study on the Anti-inflammatory Effects of Fucoidan in Relation to Quality of Life in Advanced Cancer Patients. Integr. Cancer Ther..

[99] Janjušević L., Karaman M., Šibul F., Tommonaro G., Iodice C., Jakovljević D., Pejin B. (2017). The lignicolous fungus *Trametes versicolor* (L.) Lloyd (1920): A promising natural source of antiradical and AChE inhibitory agents. J. Enzym. Inhib. Med. Chem..

[100] Wang K., Wang Z., Cui R., Chu H. (2019). Polysaccharopeptide from Trametes versicolor blocks inflammatory osteoarthritis pain-morphine tolerance effects via activating cannabinoid type 2 receptor. Int. J. Biol. Macromol..

[101] Khan M., Rahman M., Zaman S., Jahangir T.A., Razu M.H. (2015). Omega-3 Polyunsaturated Fatty Acids from Algae. Recent Adv. Microalgal Biotechnol..

[102] Zhang A.C., Singh S., Craig J.P., Downie L.E. (2020). Omega-3 fatty acids and eye health: Opinions and self-reported practice behaviors of optometrists in Australia and New Zealand. Nutrients.

[103] Pellegrini M., Senni C., Bernabei F., Cicero A.F.G., Vagge A., Maestri A., Scorcia V., Giannaccare G. (2020). The Role of Nutrition and Nutritional Supplements in Ocular Surface Diseases. Nutrients.

[104] Simopoulos A.P. (2002). The importance of the ratio of omega-6/omega-3 essential fatty acids. Biomed. Pharmacother..

[105] Downie L.E., Ng S.M., Lindsley K.B., Akpek E.K. (2019). Omega-3 and omega-6 polyunsaturated fatty acids for dry eye disease. Cochrane Database Syst. Rev..

[106] Polcz M.E., Barbul A. (2019). The Role of Vitamin A in Wound Healing. Nutr. Clin. Pract..

[107] Zasada M., Budzisz E. (2019). Retinoids: Active molecules influencing skin structure formation in cosmetic and dermatological treatments. Adv. Dermatol. Allergol. Postępy Dermatol. I Alergol..

[108] Blaner W.S., Marriott B.P., Birt D.F., Stallings V.A., Yates A.A. (2020). Chapter 5—Vitamin A and provitamin A carotenoids. Present Knowledge in Nutrition.

[109] Information N.C.F.B. PubChem Compound Summary for CID 638015, Retinal. https://pubchem.ncbi.nlm.nih.gov/compound/Retinal.

[110] Proinsias K., Giedyk M., Gryko D. (2013). Vitamin B12: Chemical modifications. Chemical Society Reviews.

[111] Romain M., Sviri S., Linton D.M., Stav I., Van Heerden P.V. (2016). The role of Vitamin B12 in the critically ill—A review. Anaesth. Intensive Care.

[112] Alonso E.R., León I., Alonso J.L. (2021). The role of the intramolecular interactions in the structural behavior of biomolecules: Insights from rotational spectroscopy. Intra- and Intermolecular Interactions between Non-Covalently Bonded Species.

[113] Macan A.M., Kraljević T.G., Raić-Malić S. (2019). Therapeutic Perspective of Vitamin C and Its Derivatives. Antioxidants.

[114] Carr A.C., Maggini S. (2017). Vitamin C and Immune Function. Nutrients.

[115] Holford P., Carr A.C., Jovic T.H., Ali S.R., Whitaker I.S., Marik P.E., Smith A.D. (2020). Vitamin C—An Adjunctive Therapy for Respiratory Infection, Sepsis and COVID-19. Nutrients.

[116] Peponis V., Papathanasiou M., Kapranou A., Magkou C., Tyligada A., Melidonis A., Droso T., Sitaras N.M. (2002). Protective role of oral antioxidant supplementation in ocular surface of diabetic patients. Br. J. Ophthalmol..

[117] Charoenngam N., Holick M.F. (2020). Immunologic Effects of Vitamin D on Human Health and Disease. Nutrients.

[118] Okamura W.H., Midland M.M., Hammond M.W., Abd Rahman N., Dormanen M.C., Nemere I., Norman A.W. (1995). Chemistry and conformation of vitamin D molecules. J. Steroid Biochem. Mol. Biol..

[119] Preedy V.R. (2015). Selenium: Chemistry, Analysis, Function and Effects.

[120] Wang N., Tan H.-Y., Li S., Xu Y., Guo W., Feng Y. (2017). Supplementation of Micronutrient Selenium in Metabolic Diseases: Its Role as an Antioxidant. Oxidative Med. Cell. Longev..

[121] Jarosz M., Olbert M., Wyszogrodzka G., Młyniec K., Librowski T. (2017). Antioxidant and anti-inflammatory effects of zinc. Zinc-dependent NF-κB signaling. Inflammopharmacology.

[122] Barak P., Helmke P.A. (1993). The Chemistry of Zinc. Zinc in Soils and Plants.

[123] Sanna A., Firinu D., Zavattari P., Valera P. (2018). Zinc Status and Autoimmunity: A Systematic Review and Meta-Analysis. Nutrients.

[124] Grahn B.H., Paterson P.G., Gottschall-Pass K.T., Zhang Z. (2001). Zinc and the Eye. J. Am. Coll. Nutr..

[125] Skrajnowska D., Bobrowska-Korczak B. (2019). Role of Zinc in Immune System and Anti-Cancer Defense Mechanisms. Nutrients.

[126] Lin P.H., Sermersheim M., Li H., Lee P.H.U., Steinberg S.M., Ma J. (2017). Zinc in Wound Healing Modulation. Nutrients.

[127] Silveira D., Prieto-Garcia J.M., Boylan F., Estrada O., Fonseca-Bazzo Y.M., Jamal C.M., Magalhães P.O., Pereira E.O., Tomczyk M., Heinrich M. (2020). COVID-19: Is There Evidence for the Use of Herbal Medicines as Adjuvant Symptomatic Therapy?. Front. Pharmacol..

[128] Pokkalath A.S., Sawant A., Sawarkar S.P. (2022). Herbal medicine for ocular diseases: An age old therapy and its future perspective. J. Drug Deliv. Sci. Technol..

[129] De Almeida Alvarenga L., Borges N.A., Moreira L.D.S.G., Resende Teixeira K.T., Carraro-Eduardo J.C., Dai L., Stenvinkel P., Lindholm B., Mafra D. (2019). Cranberries—potential benefits in patients with chronic kidney disease. Food Funct..

[130] Gbinigie O.A., Spencer E.A., Heneghan C.J., Lee J.J., Butler C.C. (2020). Cranberry Extract for Symptoms of Acute, Uncomplicated Urinary Tract Infection: A Systematic Review. Antibiotics.

[131] Krueger C.G., Reed J.D., Feliciano R.P., Howell A.B. (2013). Quantifying and characterizing proanthocyanidins in cranberries in relation to urinary tract health. Anal. Bioanal. Chem..

[132] Jepson R.G., Williams G., Craig J.C. (2012). Cranberries for preventing urinary tract infections. Cochrane Database Syst. Rev..

[133] Zhao S., Liu H., Gu L. (2020). American cranberries and health benefits—An evolving story of 25 years. J. Sci. Food Agric..

[134] Neto C.C. (2011). Cranberries: Ripe for more cancer research?. J. Sci. Food Agric..

[135] Sharma S., Sahu D., Das H.R., Sharma D. (2011). Amelioration of collagen-induced arthritis by Salix nigra bark extract via suppression of pro-inflammatory cytokines and oxidative stress. Food Chem. Toxicol..

[136] da Silva G.L., Luft C., Lunardelli A., Amaral R.H., da Silva Melo D.A., Donadio M.V.F., Nunes F.B., de Azambuja M.S., Santana J.C., Moraes C.M.B. (2015). Antioxidant, analgesic and anti-inflammatory effects of lavender essential oil. An. Acad. Bras. Ciências.

[137] Seo E., Shin Y.K., Hsieh Y.S., Lee J.M., Seol G.H. (2021). Linalyl acetate as a potential preventive agent against muscle wasting in rheumatoid arthritis rats chronically exposed to nicotine. J. Pharmacol. Sci..

[138] Aboutaleb N., Jamali H., Abolhasani M., Pazoki Toroudi H. (2019). Lavender oil (*Lavandula angustifolia*) attenuates renal ischemia/reperfusion injury in rats through suppression of inflammation, oxidative stress and apoptosis. Biomed. Pharmacother..

[139] Donelli D., Antonelli M., Bellinazzi C., Gensini G.F., Firenzuoli F. (2019). Effects of lavender on anxiety: A systematic review and meta-analysis. Phytomedicine.

[140] Srivastava P., Tiwari A. (2016). A New Insight of Herbal Promises Against Ocular Disorders: An Occuloinformatics Approach. Curr. Top. Med. Chem..

[141] Robinson P.K. (2015). Enzymes: Principles and biotechnological applications. Essays Biochem..

[142] Mazorra-Manzano M.A., Ramírez-Suarez J.C., Yada R.Y. (2018). Plant proteases for bioactive peptides release: A review. Crit. Rev. Food Sci. Nutr..

[143] Cho H.D., Kim J.H., Won Y.S., Moon K.D., Seo K.I. (2019). Inhibitory Effects of Pectinase-Treated Prunus Mume Fruit Concentrate on Colorectal Cancer Proliferation and Angiogenesis of Endothelial Cells. J. Food Sci..

[144] Kong X.Z., Guo M.M., Hua Y.F., Cao D., Zhang C.M. (2008). Enzymatic preparation of immunomodulating hydrolysates from soy proteins. Bioresour. Technol..

[145] Palma M.L., Zamith-Miranda D., Martins F.S., Bozza F.A., Nimrichter L., Montero-Lomeli M., Marques E.T.A., Douradinha B. (2015). Probiotic Saccharomyces cerevisiae strains as biotherapeutic tools: Is there room for improvement?. Appl. Microbiol. Biotechnol..

[146] Datta S., Timson D.J., Annapure U.S. (2017). Antioxidant properties and global metabolite screening of the probiotic yeast Saccharomyces cerevisiae var. boulardii. J. Sci. Food Agric..

[147] Smecuol E., Hwang H.J., Sugai E., Corso L., Cherñavsky A.C., Bellavite F.P., González A., Vodánovich F., Moreno M.L., Vázquez H. (2013). Exploratory, Randomized, Double-blind, Placebo-controlled Study on the Effects of Bifidobacterium infantis Natren Life Start Strain Super Strain in Active Celiac Disease. J. Clin. Gastroenterol..

[148] Olivares M., Castillejo G., Varea V., Sanz Y. (2014). Double-blind, randomised, placebo-controlled intervention trial to evaluate the effects of Bifidobacterium longum CECT 7347 in children with newly diagnosed coeliac disease. Br. J. Nutr..

[149] Quagliariello A., Aloisio I., Bozzi Cionci N., Luiselli D., D’Auria G., Martinez-Priego L., Pérez-Villarroya D., Langerholc T., Primec M., Mičetić-Turk D. (2016). Effect of Bifidobacterium breve on the Intestinal Microbiota of Coeliac Children on a Gluten Free Diet: A Pilot Study. Nutrients.

[150] Caio G., Riegler G., Patt Urelli M., Facchiano A., De Magistris L., Sapone A. (2017). Pathophysiology of non-celiac gluten sensitivity: Where are we now?. Minerva Gastroenterol. E Dietol..

[151] D’Arienzo R., Stefanile R., Maurano F., Mazzarella G., Ricca E., Troncone R., Auricchio S., Rossi M. (2011). Immunomodulatory Effects of Lactobacillus casei Administration in a Mouse Model of Gliadin-Sensitive Enteropathy. Scand. J. Immunol..

[152] Sergeev I.N., Aljutaily T., Walton G., Huarte E. (2020). Effects of Synbiotic Supplement on Human Gut Microbiota, Body Composition and Weight Loss in Obesity. Nutrients.

[153] Malbaša R.V., Lončar E.S., Vitas J.S., Čanadanović-Brunet J.M. (2011). Influence of starter cultures on the antioxidant activity of kombucha beverage. Food Chem..

[154] Yang B., Wang J., Tang B., Liu Y., Guo C., Yang P., Yu T., Li R., Zhao J., Zhang L. (2011). Characterization of Bioactive Recombinant Human Lysozyme Expressed in Milk of Cloned Transgenic Cattle. PLoS ONE.

[155] Cormick G., Betran A.P., Romero I.B., Cormick M.S., Belizán J.M., Bardach A., Ciapponi A. (2021). Effect of Calcium Fortified Foods on Health Outcomes: A Systematic Review and Meta-Analysis. Nutrients.

[156] Weaver C.M. (2017). Nutrition and bone health. Oral Dis..

[157] Tablante E.C., Pachón H., Guetterman H.M., Finkelstein J.L. (2019). Fortification of wheat and maize flour with folic acid for population health outcomes. Cochrane Database Syst. Rev..

[158] Assessment S.C.o.H.T. (2007). Benefits and Risks of Fortifying Flour with Folic Acid to Reduce the Risk of Neural Tube Defects: A Systematic Review.

[159] Rayman M.P. (2018). Multiple nutritional factors and thyroid disease, with particular reference to autoimmune thyroid disease. Proc. Nutr. Soc..

[160] Joussen A.M., Rohrschneider K., Reichling J., Kirchhof B., Kruse F.E. (2000). Treatment of Corneal Neovascularization with Dietary Isoflavonoids and Flavonoids. Exp. Eye Res..

[161] Wang B.Z., Zou Y., Li H., Yan H., Pan J.S., Yuan Z.L. (2005). Genistein Inhibited Retinal Neovascularization and Expression of Vascular Endothelial Growth Factor and Hypoxia Inducible Factor 1α in a Mouse Model of Oxygen-Induced Retinopathy. J. Ocul. Pharmacol. Ther..

[162] Huang R., Shi F., Lei T., Song Y., Hughes C.L., Liu G. (2007). Effect of the Isoflavone Genistein Against Galactose-Induced Cataracts in Rats. Exp. Biol. Med..

[163] Yilmaz A., Yildirim Ö., Tamer L., Öz Ö., Cinel L., Vatansever H., Değirmenci U., Kanik A., Atik U. (2005). Effects of Caffeic Acid Phenethyl Ester on Endotoxin-Induced Uveitis in Rats. Curr. Eye Res..

[164] Pittalà V., Salerno L., Romeo G., Siracusa M.A., Modica M.N., Romano G.L., Salomone S., Drago F., Bucolo C. (2015). Effects of novel hybrids of caffeic acid phenethyl ester and NSAIDs on experimental ocular inflammation. Eur. J. Pharmacol..

[165] Şahin A., Kürşat Cingü A., Kaya S., Türkcü G., Arı Ş., Evliyaoğlu O., Çınar Y., Türkcü F.M., Yüksel H., Murat M. (2013). The protective effects of caffeic acid phenethyl ester in isoniazid and ethambutol-induced ocular toxicity of rats. Cutan. Ocul. Toxicol..

[166] Zhang M., Wang L., Wen D., Ren C., Chen S., Zhang Z., Hu L., Yu Z., Tombran-Tink J., Zhang X. (2021). Neuroprotection of retinal cells by Caffeic Acid Phenylethyl Ester (CAPE) is mediated by mitochondrial uncoupling protein UCP2. Neurochem. Int..

[167] Katz J.A., Karpecki P.M., Dorca A., Chiva-Razavi S., Floyd H., Barnes E., Wuttke M., Donnenfeld E. (2021). Presbyopia—A Review of Current Treatment Options and Emerging Therapies. Clin. Ophthalmol..

[168] Sharma G., Chiva-Razavi S., Viriato D., Naujoks C., Patalano F., Bentley S., Findley A., Johnson C., Arbuckle R., Wolffsohn J. (2020). Patient-reported outcome measures in presbyopia: A literature review. BMJ Open Ophthalmol.

[169] Kono K., Shimizu Y., Takahashi S., Matsuoka S., Yui K. (2014). Effect of Multiple Dietary Supplement Containing Lutein,  Astaxanthin, Cyanidin-3-Glucoside, and DHA on Accommodative Ability. Curr. Med. Chem..

[170] Horng C.-T., Ma J.-W., Shieh P.-C. (2021). Improvement of Presbyopia Using a Mixture of Traditional Chinese Herbal Medicines, Including Cassiae Semen, Wolfberry, and Dendrobium huoshanense. Evid. Based Complement. Altern. Med..

[171] Grzybowski A., Markeviciute A., Zemaitiene R. (2020). A Review of Pharmacological Presbyopia Treatment. Asia Pac. J. Ophthalmol..

[172] Kaur A., Gupta V., Christopher A.F., Malik M.A., Bansal P. (2017). Nutraceuticals in prevention of cataract—An evidence based approach. Saudi J. Ophthalmol..

[173] Braakhuis A.J., Donaldson C.I., Lim J.C., Donaldson P.J. (2019). Nutritional Strategies to Prevent Lens Cataract: Current Status and Future Strategies. Nutrients.

[174] Lee C.M., Afshari N.A. (2017). The global state of cataract blindness. Curr. Opin. Ophthalmol..

[175] Hejtmancik J.F., Shiels A. (2015). Overview of the Lens. Prog. Mol. Biol. Transl. Sci..

[176] Wei L., Liang G., Cai C., Lv J. (2016). Association of vitamin C with the risk of age-related cataract: A meta-analysis. Acta Ophthalmol..

[177] Mares-Perlman J.A., Lyle B.J., Klein R., Fisher A.I., Brady W.E., VandenLangenberg G.M., Trabulsi J.N., Palta M. (2000). Vitamin supplement use and incident cataracts in a population-based study. Arch. Ophthalmol..

[178] Robertson J.M., Donner A.P., Trevithick J.R. (1991). A possible role for vitamins C and E in cataract prevention. Am. J. Clin. Nutr..

[179] Vitale S., West S., Hallfrisch J., Alston C., Wang F., Moorman C., Muller D., Singh V., Taylor H.R. (1993). Plasma antioxidants and risk of cortical and nuclear cataract. Epidemiology.

[180] Weikel K.A., Garber C., Baburins A., Taylor A. (2014). Nutritional modulation of cataract. Nutr. Rev..

[181] Jacques P.F., Chylack L.T., Hankinson S.E., Khu P.M., Rogers G., Friend J., Tung W., Wolfe J.K., Padhye N., Willett W.C. (2001). Long-term nutrient intake and early age-related nuclear lens opacities. Arch. Ophthalmol..

[182] Leske M.C., Chylack L.T., Wu S.Y. (1991). The Lens Opacities Case-Control Study. Risk factors for cataract. Arch. Ophthalmol..

[183] Valero M.P., Fletcher A.E., De Stavola B.L., Vioque J., Alepuz V.C. (2002). Vitamin C is associated with reduced risk of cataract in a Mediterranean population. J. Nutr..

[184] Ravindran R.D., Vashist P., Gupta S.K., Young I.S., Maraini G., Camparini M., Jayanthi R., John N., Fitzpatrick K.E., Chakravarthy U. (2011). Inverse association of vitamin C with cataract in older people in India. Ophthalmology.

[185] Klein B.E., Knudtson M.D., Lee K.E., Reinke J.O., Danforth L.G., Wealti A.M., Moore E., Klein R. (2008). Supplements and age-related eye conditions the beaver dam eye study. Ophthalmology.

[186] Dherani M., Murthy G.V., Gupta S.K., Young I.S., Maraini G., Camparini M., Price G.M., John N., Chakravarthy U., Fletcher A.E. (2008). Blood levels of vitamin C, carotenoids and retinol are inversely associated with cataract in a North Indian population. Investig. Ophthalmol. Vis. Sci..

[187] Berendschot T.T., Broekmans W.M., Klopping-Ketelaars I.A., Kardinaal A.F., Van Poppel G., Van Norren D. (2002). Lens aging in relation to nutritional determinants and possible risk factors for age-related cataract. Arch. Ophthalmol..

[188] Rodriguez-Rodriguez E., Ortega R.M., Lopez-Sobaler A.M., Aparicio A., Bermejo L.M., Marin-Arias L.I. (2006). The relationship between antioxidant nutrient intake and cataracts in older people. Int. J. Vitam. Nutr. Res..

[189] Karppi J., Laukkanen J.A., Kurl S. (2012). Plasma lutein and zeaxanthin and the risk of age-related nuclear cataract among the elderly Finnish population. Br. J. Nutr..

[190] Christen W.G., Liu S., Glynn R.J., Gaziano J.M., Buring J.E. (2008). Dietary carotenoids, vitamins C and E, and risk of cataract in women: A prospective study. Arch. Ophthalmol..

[191] Moeller S.M., Voland R., Tinker L., Blodi B.A., Klein M.L., Gehrs K.M., Johnson E.J., Snodderly D.M., Wallace R.B., Chappell R.J. (2008). Associations between age-related nuclear cataract and lutein and zeaxanthin in the diet and serum in the Carotenoids in the Age-Related Eye Disease Study, an Ancillary Study of the Women’s Health Initiative. Arch. Ophthalmol..

[192] Chylack L.T., Brown N.P., Bron A., Hurst M., Kopcke W., Thien U., Schalch W. (2002). The Roche European American Cataract Trial (REACT): A randomized clinical trial to investigate the efficacy of an oral antioxidant micronutrient mixture to slow progression of age-related cataract. Ophthalmic Epidemiol..

[193] Rouen P.A., White M.L. (2018). Dry eye disease: Prevalence, assessment, and management. Home Healthc. Now.

[194] Zemanová M. (2021). Dry eye disease. A review. Ceska a Slovenska Oftalmologie: Casopis Ceske Oftalmologicke Spolecnosti a Slovenske Oftalmologicke Spolecnosti.

[195] Messmer E.M. (2015). The Pathophysiology, Diagnosis, and Treatment of Dry Eye Disease. Dtsch. Ärzteblatt Int..

[196] Eghrari A.O., Riazuddin S.A., Gottsch J.D. (2015). Overview of the Cornea: Structure, Function, and Development. Prog. Mol. Biol. Transl. Sci..

[197] Chhadva P., Goldhardt R., Galor A. (2017). Meibomian Gland Disease: The Role of Gland Dysfunction in Dry Eye Disease. Ophthalmology.

[198] Gipson I.K. (2007). The Ocular Surface: The Challenge to Enable and Protect Vision: The Friedenwald Lecture. Investig. Ophthalmol. Vis. Sci..

[199] Navarro-Partida J., Rodrigo Castro-Castaneda C., Santa Cruz-Pavlovich F.J., Abraham Aceves-Franco L., Ori Guy T., Santos A., Cruz-Pavlovich S., Lipid-Based Nanocarriers A., Lopes C.M., Lucio M. (2021). Lipid-Based Nanocarriers as Topical Drug Delivery Systems for Intraocular Diseases. Pharmaceutics.

[200] Stern M.E., Gao J., Siemasko K.F., Beuerman R.W., Pflugfelder S.C. (2004). The role of the lacrimal functional unit in the pathophysiology of dry eye. Exp. Eye Res..

[201] Baudouin C., Aragona P., Messmer E.M., Tomlinson A., Calonge M., Boboridis K.G., Akova Y.A., Geerling G., Labetoulle M., Rolando M. (2013). Role of Hyperosmolarity in the Pathogenesis and Management of Dry Eye Disease: Proceedings of the OCEAN Group Meeting. Ocul. Surf..

[202] Chatterjee S., Agrawal D., Chaturvedi P. (2021). Ocular Surface Disease Index©and the five-item dry eye questionnaire: A comparison in Indian patients with dry eye disease. Indian J. Ophthalmol..

[203] Dibajnia P., Mohammadinia M., Moghadasin M., Amiri M.A. (2012). Tear Film Break-up Time in Bipolar Disorder. Iran. J. Psychiatry.

[204] Brott N.R., Ronquillo Y. (2021). Schirmer Test. Encyclopedia of Ophthalmology.

[205] Chi S.C., Tuan H.I., Kang Y.N. (2019). Effects of Polyunsaturated Fatty Acids on Nonspecific Typical Dry Eye Disease: A Systematic Review and Meta-Analysis of Randomized Clinical Trials. Nutrients.

[206] Giannaccare G., Pellegrini M., Sebastiani S., Bernabei F., Roda M., Taroni L., Versura P., Campos E.C. (2019). Efficacy of Omega-3 Fatty Acid Supplementation for Treatment of Dry Eye Disease: A Meta-Analysis of Randomized Clinical Trials. Cornea.

[207] Bhargava R., Chandra M., Bansal U., Singh D., Ranjan S., Sharma S. (2016). A Randomized Controlled Trial of Omega 3 Fatty Acids in Rosacea Patients with Dry Eye Symptoms. Curr. Eye Res..

[208] Bhargava R., Kumar P. (2015). Oral omega-3 fatty acid treatment for dry eye in contact lens wearers. Cornea.

[209] Bhargava R., Kumar P., Arora Y. (2016). Short-Term Omega 3 Fatty Acids Treatment for Dry Eye in Young and Middle-Aged Visual Display Terminal Users. Eye Contact Lens Sci. Clin. Pract..

[210] Bhargava R., Kumar P., Kumar M., Mehra N., Mishra A. (2013). A randomized controlled trial of omega-3 fatty acids in dry eye syndrome. Int. J. Ophthalmol..

[211] Bhargava R., Kumar P., Phogat H., Kaur A., Kumar M. (2015). Oral omega-3 fatty acids treatment in computer vision syndrome related dry eye. Contact Lens Anterior Eye.

[212] Epitropoulos A.T., Donnenfeld E.D., Shah Z.A., Holland E.J., Gross M., Faulkner W.J., Matossian C., Lane S.S., Toyos M., Bucci F.A. (2016). Effect of oral re-esterified Omega-3 nutritional supplementation on dry eyes. Cornea.

[213] The Dry Eye Assessment and Management Study Research Group (2018). n−3 Fatty Acid Supplementation for the Treatment of Dry Eye Disease. N. Engl. J. Med..

[214] Kawakita T., Kawabata F., Tsuji T., Kawashima M., Shimmura S., Tsubota K. (2013). Effects of dietary supplementation with fish oil on dry eye syndrome subjects: Randomized controlled trial. Biomed. Res..

[215] Deinema L.A., Vingrys A.J., Wong C.Y., Jackson D.C., Chinnery H.R., Downie L.E. (2017). A Randomized, Double-Masked, Placebo-Controlled Clinical Trial of Two Forms of Omega-3 Supplements for Treating Dry Eye Disease. Ophthalmology.

[216] Chinnery H.R., Naranjo Golborne C., Downie L.E. (2017). Omega-3 supplementation is neuroprotective to corneal nerves in dry eye disease: A pilot study. Ophthalmic Physiol. Opt..

[217] Rand A.L., Asbell P.A. (2011). Nutritional supplements for dry eye syndrome. Curr. Opin. Ophthalmol..

[218] Alanazi S.A., El-Hiti G.A., Al-Baloud A.A., Alfarhan M.I., Al-Shahrani A., Albakri A.A., Alqahtani S., Masmali A.M. (2019). Effects of short-term oral vitamin A supplementation on the ocular tear film in patients with dry eye. Clin. Ophthalmol..

[219] Sheppard J.D., Singh R., McClellan A.J., Weikert M.P., Scoper S.V., Joly T.J., Whitley W.O., Kakkar E., Pflugfelder S.C. (2013). Long-term Supplementation With n-6 and n-3 PUFAs Improves Moderate-to-Severe Keratoconjunctivitis Sicca: A Randomized Double-Blind Clinical Trial. Cornea.

[220] Yamashita S.I., Suzuki N., Yamamoto K., Iio S.I., Yamada T. (2019). Effects of MaquiBright((R)) on improving eye dryness and fatigue in humans: A randomized, double-blind, placebo-controlled trial. J. Tradit. Complement. Med..

[221] Hyon J.Y., Han S.B. (2022). Dry Eye Disease and Vitamins: A Narrative Literature Review. Appl. Sci..

[222] Kador P.F. (2015). Antioxidant Eye Drops.

[223] Fogagnolo P., Quisisana C., Caretti A., Marchina D., Dei Cas M., Melardi E., Rossetti L. (2020). Efficacy and Safety of VisuEvo((R)) and Cationorm((R)) for the Treatment of Evaporative and Non-Evaporative Dry Eye Disease: A Multicenter, Double-Blind, Cross-Over, Randomized Clinical Trial. Clin. Ophthalmol..

[224] Allison K., Patel D., Alabi O. (2020). Epidemiology of Glaucoma: The Past, Present, and Predictions for the Future. Cureus.

[225] Weinreb R.N., Aung T., Medeiros F.A. (2014). The Pathophysiology and Treatment of Glaucoma: A Review. JAMA.

[226] Vetrugno M., Uva M.G., Russo V., Iester M., Ciancaglini M., Brusini P., Centofanti M., Rossetti L.M. (2012). Oral Administration of Forskolin and Rutin Contributes to Intraocular Pressure Control in Primary Open Angle Glaucoma Patients Under Maximum Tolerated Medical Therapy. J. Ocular Pharmacol. Ther..

[227] Mutolo M.G., Albanese G., Rusciano D., Pescosolido N. (2016). Oral Administration of Forskolin, Homotaurine, Carnosine, and Folic Acid in Patients with Primary Open Angle Glaucoma: Changes in Intraocular Pressure, Pattern Electroretinogram Amplitude, and Foveal Sensitivity. J. Ocular Pharmacol. Ther..

[228] Romeo Villadóniga S., Rodríguez García E., Sagastagoia Epelde O., Álvarez Díaz M.D., Domingo Pedrol J.C. (2018). Effects of Oral Supplementation with Docosahexaenoic Acid (DHA) plus Antioxidants in Pseudoexfoliative Glaucoma: A 6-Month Open-Label Randomized Trial. J. Ophthalmol..

[229] Galbis-Estrada C., Pinazo-Durán M.D., Cantú-Dibildox J., Marco-Ramírez C., Díaz-Llópis M., Benítez-del-Castillo J. (2013). Patients undergoing long-term treatment with antihypertensive eye drops responded positively with respect to their ocular surface disorder to oral supplementation with antioxidants and essential fatty acids. Clin. Interv. Aging.

[230] Bonyadi M.H.J., Yazdani S., Saadat S. (2014). The ocular hypotensive effect of saffron extract in primary open angle glaucoma: A pilot study. BMC Complement. Altern. Med..

[231] Sim R.H., Sirasanagandla S.R., Das S., Teoh S.L. (2022). Treatment of Glaucoma with Natural Products and Their Mechanism of Action: An Update. Nutrients.

[232] Ohguro H., Ohguro I., Katai M., Tanaka S. (2012). Two-year randomized, placebo-controlled study of black currant anthocyanins on visual field in glaucoma. Ophthalmologica.

[233] Parisi V., Centofanti M., Ziccardi L., Tanga L., Michelessi M., Roberti G., Manni G. (2015). Treatment with citicoline eye drops enhances retinal function and neural conduction along the visual pathways in open angle glaucoma. Graefe’s Arch. Clin. Exp. Ophthalmol..

[234] Parisi V., Centofanti M., Gandolfi S., Marangoni D., Rossetti L., Tanga L., Tardini M., Traina S., Ungaro N., Vetrugno M. (2014). Effects of coenzyme Q10 in conjunction with vitamin e on retinal-evoked and cortical-evoked responses in patients with open-angle glaucoma. J. Glaucoma.

[235] Kassoff A., Kassoff J., Buehler J., Eglow M., Kaufman F., Mehu M., Kieval S., Mairs M., Graig B., Quattrocchi A. (2001). A Randomized, Placebo-Controlled, Clinical Trial of High-Dose Supplementation With Vitamins C and E, Beta Carotene, and Zinc for Age-Related Macular Degeneration and Vision Loss: AREDS Report No. 8. Arch. Ophthalmol..

[236] Chew E.Y., Clemons T.E., SanGiovanni J.P., Danis R.P., Ferris F.L., Elman M.J., Antoszyk A.N., Ruby A.J., Orth D., Bressler S.B. (2014). Secondary Analyses of the Effects of Lutein/Zeaxanthin on Age-Related Macular Degeneration Progression: AREDS2 Report No. 3. JAMA Ophthalmol..

[237] Beatty S., Chakravarthy U., Nolan J.M., Muldrew K.A., Woodside J.V., Denny F., Stevenson M.R. (2013). Secondary outcomes in a clinical trial of carotenoids with coantioxidants versus placebo in early age-related macular degeneration. Ophthalmology.

[238] Berrow E.J., Bartlett H.E., Eperjesi F., Gibson J.M. (2013). The effects of a lutein-based supplement on objective and subjective measures of retinal and visual function in eyes with age-related maculopathy—A randomised controlled trial. Br. J. Nutr..

[239] Ma L., Liu R., Du J.H., Liu T., Wu S.S., Liu X.H. (2016). Lutein, Zeaxanthin and Meso-zeaxanthin Supplementation Associated with Macular Pigment Optical Density. Nutrients.

[240] Weigert G., Kaya S., Pemp B., Sacu S., Lasta M., Werkmeister R.M., Dragostinoff N., Simader C., Garhöfer G., Schmidt-Erfurth U. (2011). Effects of Lutein Supplementation on Macular Pigment Optical Density and Visual Acuity in Patients with Age-Related Macular Degeneration. Investig. Ophthalmol. Vis. Sci..

[241] Bovier E.R., Hammond B.R. (2015). A randomized placebo-controlled study on the effects of lutein and zeaxanthin on visual processing speed in young healthy subjects. Arch. Biochem. Biophys..

[242] García-Layana A., Recalde S., Alamán A.S., Robredo P.F. (2013). Effects of Lutein and Docosahexaenoic Acid Supplementation on Macular Pigment Optical Density in a Randomized Controlled Trial. Nutrients.

[243] Allegrini D., Raimondi R., Angi M., Ricciardelli G., Montericcio A., Borgia A., Romano M.R. (2021). Curcuma-Based Nutritional Supplement in Patients with Neovascular Age-Related Macular Degeneration. J. Med. Food.

[244] Matos A.L., Bruno D.F., Ambrósio A.F., Santos P.F. (2020). The Benefits of Flavonoids in Diabetic Retinopathy. Nutrients.

[245] Moschos M.M., Dettoraki M., Tsatsos M., Kitsos G., Kalogeropoulos C. (2017). Effect of carotenoids dietary supplementation on macular function in diabetic patients. Eye Vis..

[246] Zhang P.C., Wu C.R., Wang Z.L., Wang L.Y., Han Y., Sun S.L., Li Q.S., Ma L. (2017). Effect of lutein supplementation on visual function in nonproliferative diabetic retinopathy. Asia Pac. J. Clin. Nutr..

[247] Hu B.J., Hu Y.N., Lin S., Ma W.J., Li X.R. (2011). Application of Lutein and Zeaxanthin in nonproliferative diabetic retinopathy. Int. J. Ophthalmol..

[248] Domanico D., Fragiotta S., Cutini A., Carnevale C., Zompatori L., Vingolo E.M. (2015). Circulating levels of reactive oxygen species in patients with nonproliferative diabetic retinopathy and the influence of antioxidant supplementation: 6-month follow-up. Indian J. Ophthalmol..

[249] Korenfeld M.S., Robertson S.M., Stein J.M., Evans D.G., Rauchman S.H., Sall K.N., Venkataraman S., Chen B.L., Wuttke M., Burns W. (2021). Topical lipoic acid choline ester eye drop for improvement of near visual acuity in subjects with presbyopia: A safety and preliminary efficacy trial. Eye.

[250] Kangari H., Eftekhari M.H., Sardari S., Hashemi H., Salamzadeh J., Ghassemi-Broumand M., Khabazkhoob M. (2013). Short-term Consumption of Oral Omega-3 and Dry Eye Syndrome. Ophthalmology.

[251] Teoh S.L., Ngorsuraches S., Lai N.M., Chaiyakunapruk N. (2021). Consumer Preferences and Willingness to Pay for Nutraceuticals: A Discrete Choice Experiment. Value Health Reg. Issues.

